# A Thermodynamically Consistent Model of Quasibrittle Elastic Damaged Materials Based on a Novel Helmholtz Potential and Dissipation Function

**DOI:** 10.3390/ma14216323

**Published:** 2021-10-23

**Authors:** Inez Kamińska, Aleksander Szwed

**Affiliations:** Faculty of Civil Engineering, Warsaw University of Technology, 00-637 Warsaw, Poland; a.szwed@il.pw.edu.pl

**Keywords:** Helmholtz energy, dissipation, damage, damage parameters, thermodynamic consistency, concrete, quasibrittle materials, bimodular elasticity

## Abstract

In the paper, a thermodynamically consistent model of elastic damaged material in the framework of small strain theory is formulated, describing the process of deterioration in quasibrittle materials, concrete in particular. The main goal is to appropriately depict the distinction between material responses in tension and compression. A novel Helmholtz energy and a dissipation potential including three damage parameters are introduced. The Helmholtz function has a continuous first derivative with respect to strain tensor. Based on the assumed functions, the strain–stress relationship, the damage condition, the evolution laws, and the tangent stiffness tensor are derived. The model’s predictions for uniaxial tension, uniaxial compression, uniaxial cyclic compression–tension, and pure shear tests are calculated using Wolfram Mathematica in order to identify the main features of the model and to grasp the physical meaning of an isotropic damage parameter, a tensile damage parameter, and a compressive damage parameter. Their values can be directly bound to changes of secant stiffness and generalized Poisson’s ratio. An interpretation of damage parameters in association with three mechanisms of damage is given. The considered dissipation potential allows a flexible choice of a damage condition. The influence of material parameters included in dissipation function on damage mode interaction is discussed.

## 1. Introduction

Many microscopic cavities, microcracks, flaws, and so forth can be observed on the microscale in solid materials. Those defects owe their origins to disturbances in the interatomic or intermolecular bonds. The technological casting process or loading leads to the development of existing micro- and mesoscale structural irregularities as well as to the initiation of new breakages in material structure. The evolution of those defects causes fracture of a material together with deterioration of its mechanical properties called a damage phenomenon. Damage can occur on the atomic scale, microscale, and mesoscale, leading to macroscale effects. The physical mechanisms of damage include cleavage (decohesion), growth, and coalescence of microvoids, glide plane decohesion, and grain boundary decohesion. Deterioration of mechanical properties mainly depends on the type of material and loading conditions. Ductile damage can be observed in metallic materials, while brittle damage appears in rocks, concrete, ceramics, and composite materials. At an elevated temperature, polycrystalline metals exhibit creep damage. Majority of materials deteriorate due to cyclic loading. Damage and fracture processes ongoing in a material treated as a macroscopic material continuum are successfully described in the framework of continuum damage mechanics [1,2,3,4].

The growing complexity of structures demands improvements of existing constitutive models of quasibrittle materials, concrete in particular. An accurate description of their behavior is a subject of an ongoing research [5,6,7,8,9,10,11,12,13,14,15,16]. Designing complex and innovative structures requires a sound understanding of the mechanical behavior of concrete. One of the crucial characteristics of concrete (and many other quasibrittle materials) is its low tensile strength, which allows tensile cracking at very low stresses compared with stresses in the compression range. This tensile cracking significantly reduces the stiffness of structural elements. Nucleation, growth, and coalescence of microcracks present in concrete lead to degradation of stiffness and irreversible deformations. Therefore, advanced and accurate constitutive modeling of concrete under complex loading conditions is of keen interest from the point of view of rational prediction of damage and failure.

In phenomenological constitutive modeling, two main aspects are of utmost importance: the thermodynamic consistency of the model and its compatibility with experimental data [6,13,17,18,19,20,21,22]. In the article, we are predominately focused on the first aspect, although we reference to basic experiments as well. In the framework of small strain, we propose a model of an elastic damaged material based on novel Helmholtz and dissipation functions that both satisfy the laws of thermodynamics and capture the crucial difference between tensile and compressive behavior of materials. The fundamentals of thermodynamically consistent constitutive modeling based on two potentials were laid in the last four decades and have well consolidated since [1,2,7,14,17,18,19,20]. The particulars of the framework for geotechnical materials have been improved upon [19,20], but the actual usage is limited due to mathematical difficulties, especially performing the Fenchel–Legendre transformation, although some examples are present in the appropriate literature [6,7,8,9,16,21]. In the paper, we introduce two potentials: a Helmholtz free energy and a dissipation and use the discussed above approach to derive relations that can be directly applied in numeric calculations and do not diverge from the structure of commonly used models.

Quasibrittle materials are characterized by two features: failure is caused mainly by fracture rather than plastic yield, and “the fracture front is surrounded by a large fracture-process zone in which progressive distributed cracking or other damage takes place” [5]. We focus on concrete as the main representative of quasibrittle materials’ class extensively used in civil engineering, but the regarded setting also includes rocks [19] and some alloys [23,24]. In the rough approximation, the degradation of its properties can be seen as isotropic and thus described by one damage parameter, d, which reflects the decrease in Young’s modulus (or an actual decrease in a cross section carrying loading). As a result, the Helmholtz function is the strain energy of an isotropic body multiplied by factor 1−d [1,2,3]. However, concrete subjected to a sufficiently high level of loading exhibits major differences between the behavior in tension and compression due to complex interactions between its components [25,26,27,28,29,30,31,32]. Therefore, at least two different damage parameters are needed, connected to positive and negative parts of the strain (or stress) tensor [33]. This idea is widely used (see, for example, [1,2,4,11,33,34,35,36]) and well grounded in experiments, but it is cumbersome to employ it in the numerical calculations due to the discontinuous relation between the stress tensor and the strain tensor for the uniaxial tests. Among other approaches, there are concepts to use the fully anisotropic models [10,13,15,16,21,22,37], to bind the Helmholtz function with the direction of emerging cracks [9], or to connect damage parameters to the split of energy into bulk and distortional parts [4]. We make use of the last idea but introduce three damage parameters instead of two: one describing isotropic damage (d) and two other, bound to the volumetric part of the Helmholtz function, separating the degradation into tension ($d_{T}$) and compression ($d_{C}$). This assumption allows for describing the basic features of concrete’s behavior.

Evolution laws of damage parameters are often making degradation growth dependent on parts of strain energy [1,2], while damage conditions are similar or, in models including plasticity, the same as yield conditions [13,22,34,35,36,38,39]. We propose a dissipation function that allows for including those key features. The resultant damage condition conjugated to the introduced dissipation is the one presented in [40], inspired by the Ottosen’s shape function [41].

After this introduction, the paper is organized as follows. As a starting point, a brief summary of the framework of constitutive modeling of thermodynamically consistent rate-independent materials based on [7,14,19,20] is given in Section 2. In the main part of the paper (Section 3), we introduce two potentials: a dissipation and a novel Helmholtz function. We employ three scalar damage parameters, which improves the model’s flexibility and allows for overcoming differentiability issues. Next, using the adopted framework, we derive all the relations necessary for the numerical implementation. All material parameters included in the damage condition are given by closed analytical formulae. Then, in Section 4 we show the results of calculations for simple tests: the uniaxial compression, uniaxial tension, uniaxial cyclic tension–compression, and simple shear, which allow for understanding the meaning of the introduced damage parameters and parameters involved in the dissipation potential. The presented outcomes are obtained by direct numerical solving of sets of the model differential equations: calculations are performed using Wolfram Mathematica. In Section 5, we interpret three damage parameters in reference to the physical course of deterioration. The influence of material parameters, included in the definition of dissipation function, on damage mode interaction is discussed. The most relevant research outcomes and conclusions are drawn in Section 6.

## 2. Framework for Thermodynamically Consistent Constitutive Modeling of Elastic Damaged Materials Using Helmholtz Energy and Dissipation Function

A thermodynamically consistent material model is a model that ensures the satisfaction of the first and the second law of thermodynamics in the form of the Clausius–Duhem inequality [18]. Basically, it states that dissipation in any real process, reversible or not, has to be non-negative. In order to fulfill this condition, using an approach described in [7,14,19,20], we have to obey the below-described algorithm of consecutive steps in the design of a material’s model.

The employed framework for elastic-plastic materials is described in [19,20] with extension to damaged materials in [7,14]. It is quite general and allows any of four energy potentials (Helmholtz or Gibbs or internal energy or enthalpy) and either dissipation or damage condition to be the initial points of the formulation. It admits tensorial internal damage variables. The presented version of the framework is reduced to suit the considered problem: the equations are narrowed down to the case of elasticity and damage described by a Helmholtz potential and a dissipation function dependent on multiple scalar damage parameters. Based on this kinematic description of damage, the goal is to obtain the following equations: a strain–stress relationship, a damage condition, evolution equations, and a definition of the fourth-order stiffness tensor of an elastic damaged material.

The first and the most difficult step of the scheme is to assume two appropriate functions: a Helmholtz potential and a dissipation. This requires careful consideration regarding both mathematical properties, such as convexity, and the ability to describe the real behavior of material.

The Helmholtz energy $H\left(\varepsilon ,d_{i}\right)$ must be a function that is strictly convex with respect to strain tensor ε, convex with respect to damage parameters $d_{i}$ (i=1,2,..,N, where N is the number of damage parameters in a considered model), and strictly non-negative with respect to ε, meaning that it is null for ε=0 and positive elsewhere. On the other hand, dissipation $D\left({\dot{d}}_{i}\right)$ is a convex, strictly non-negative, and, for rate-independent materials, a homogenous of degree 1 function of rates, ${\dot{d}}_{i}\left(t\right)$, where t is time. It can also have non-rate-type arguments; for example, when hardening is involved, it can additionally depend on the current values of damage parameters $D\left({\dot{d}}_{i};d_{i}\right)$ (compare in [7,14,19,20]).

Now, the Cauchy stress tensor σ and the generalized stresses $\sigma_{di}$ can be calculated, differentiating the Helmholtz potential:

(1) $$\sigma =\frac{\partial H}{\partial \varepsilon },\;\;\sigma_{di}=-\frac{\partial H}{\partial d_{i}}$$

and next, the dissipative stresses related to the dissipation function can be obtained using the following formula:

(2) $${\bar{\sigma }}_{di}=\frac{\partial D}{\partial {\dot{d}}_{i}}.$$

Dissipation, being a homogenous function of degree 1, satisfies the suitable Euler’s theorem of the form:

(3) $$D\left({\dot{d}}_{i}\right)=\sum_{i=1}^{N}\frac{\partial H}{\partial {\dot{d}}_{i}}{\dot{d}}_{i}=\sum_{i=1}^{N}{\bar{\sigma }}_{di}{\dot{d}}_{i}.$$

As a result, the following Fenchel–Legendre transformation binds the dissipation and the conjugated damage function $\bar{F}\left({\bar{\sigma }}_{di}\right)$:

(4) $$D\left({\dot{d}}_{i}\right)+\lambda \;\bar{F}\left({\bar{\sigma }}_{di}\right)=\sum_{i=1}^{N}{\bar{\sigma }}_{di}{\dot{d}}_{i}.$$

Like in the plastic flow theory, this is equivalent to:

(5) $$\lambda \;\bar{F}\left({\bar{\sigma }}_{di}\right)=0\;\mathrm{and}\;\lambda \geq 0\;\mathrm{and}\;\bar{F}\left({\bar{\sigma }}_{di}\right)\leq 0,$$

λ being a damage multiplier. In turn, if degradation is evolving, the following damage condition occurs:

(6) $$\bar{F}\left({\bar{\sigma }}_{di}\right)=0$$

and λ>0 during the purely elastic process, λ=0, and $\bar{F}\left({\bar{\sigma }}_{di}\right)<0$. The damage condition (6) needs to be complemented by the consistency condition:

(7) $$\dot{\bar{F}}\left({\bar{\sigma }}_{di}\right)=0,$$

where the upper dot denotes derivative with respect to time. The laws of evolution of damage parameters are associated with Equation (6), that is,

(8) $${\dot{d}}_{i}=\lambda \frac{\partial \;\bar{F}}{\partial \;{\bar{\sigma }}_{di}}.$$

It needs to be stressed that, in general, performing transformation described by Equation (4) is not a trivial task. In this paper, we will use a quite simple dissipation function, but getting the conjugated function, still, is not an “automatic” process.

At this point, we have not established a connection between the two potentials yet. So far, we have defined two independent sets of the generalized stresses in Equation (1) resulting from the Helmholtz energy and the dissipative stresses in Equation (2) based on the dissipation function. The role of the connector is played by the orthogonality principle of Ziegler [17,19,20]. The principle is based on equalization of the dissipation due to the rate of the Helmholtz energy and the proposed dissipation potential. Consequently, it states that the generalized and the respective dissipative stresses are equal:

(9) $${\bar{\sigma }}_{di}=\sigma_{di}.$$

Equation (9) is a categorizing hypothesis: it suits a wide class of engineering materials (compare in [19]). The presented framework for constitutive modelling gives enough flexibility in the selection of appropriate Helmholtz potential and dissipation function.

To derive the incremental form of the constitutive relationships and calibrate the model, it is convenient to transform the above obtained equations to the Cauchy stress space. As Equation (9) is in force, we can express the damage condition (Equation (6)), the consistency condition (Equation (7)), and the evolution laws (Equation (8)) via the generalized stresses and, in turn through Equation (1), via the Cauchy stress tensor and damage parameters, that is,

(10) $$F\left(\sigma \right)=0,\;\;\dot{F}\left(\sigma \right)=0,\;{\dot{d}}_{i}=\lambda \;f_{i}\left(\sigma \right).$$

In general, case functions $f_{i}$ are not bound directly to F (are not its derivatives), since in the space of generalized stresses, the evolution laws do not have to be associated.

Hardening can be easily incorporated by making material constants dependent on the current values of damage parameters, which will be discussed briefly in the subsequent sections.

For numerical implementation, it is a vital part of the formulation to provide components of the fourth-order tensor of tangent stiffness. The procedure is classic: from the consistency condition included in Equation (10), we obtain multiplier λ and incorporate it in the formula for stress increment. The details are given in [18,20].

## 3. Formulation of Elastic Damaged Material Model

In this section, a model of an elastic damaged material is presented. We propose two novel governing functions—a Helmholtz potential and a dissipation—and obtain all the useful relations following the steps described in Section 2.

### 3.1. Helmholtz Energy

Let us define the cylindrical invariants of the strain tensor:

(11) $$p=\frac{1}{\sqrt{3}}\mathrm{tr}\varepsilon ,\;q=\sqrt{\mathrm{tr}e^{2}},$$

where tr denotes the trace of a second-order tensor and $e=\varepsilon -\frac{1}{3}\left(\mathrm{tr}\varepsilon \right)I$ is the strain tensor’s deviator, I being the second-order unit tensor. Now we can introduce the following Helmholtz function:

(12) $$H\left(\varepsilon ,d,d_{T},d_{C}\right)=\left(1-d\right)\left[\frac{3}{2}K_{M}\left(d_{T},d_{C}\right)p^{2}+\frac{3}{2}K_{D}\left(d_{T},d_{C}\right)p\sqrt{p^{2}}+G\;q^{2}\right].$$

0≤d<1 is called an isotropic damage parameter, G denotes the initial shear modulus (the shear modulus of an isotropic undamaged material), while $K_{M}\left(d_{T},d_{C}\right)$ and $K_{D}\left(d_{T},d_{C}\right)$ are partial bulk moduli dependent on the tensile damage parameter $0\leq d_{T}<1$ and the compressive damage parameter $0\leq d_{C}<1$:

(13) $$G=\frac{E}{2\left(1+\nu \right)},\;\;K_{M}\left(d_{T},d_{C}\right)=\frac{E}{6}\left[\frac{1-d_{T}}{1-2\nu +2\;d_{T}\left(1+\nu \right)}+\frac{1-d_{C}}{1-2\nu +2\;d_{C}\left(1+\nu \right)}\right],\;\;K_{D}\left(d_{T},d_{C}\right)=\frac{E\left(d_{C}-d_{T}\right)}{2\left[1-2\nu +2\;d_{T}\left(1+\nu \right)\right]\left[1-2\nu +2\;d_{C}\left(1+\nu \right)\right]}.$$

The tensile damage parameter $d_{T}$ is linked to the uniaxial tension, while the compressive damage parameter $d_{C}$ is related to the uniaxial compression. E>0 is the initial Young’s modulus of an isotropic undamaged material, and ν is the respective Poisson’s ratio.

Let us emphasize that the proposed Helmholtz energy is an isotropic and positive homogeneous function of degree 2 with respect to the strain tensor: $H\left(\beta \varepsilon \right)=\beta^{2}H\left(\varepsilon \right)$ for any β>0. In general, this property will result in a nonlinear constitutive relationship; in the particular case of the usually used quadratic form of H, we obtain a linear relationship. Two first terms in Equation (12) describe bulk energy, while the last one constitutes the distortional part. Potential expressed by Equation (12) satisfies all the requirements described in Section 2, that is, convexity and non-negativity. The introduction of the term $p\sqrt{p^{2}}$ allows for obtaining a continuous first derivative with respect to ε, which implicates the existence of a continuous relation between the stress and the strain tensors. Denoting:

(14) $$K_{T}\left(d_{T}\right)=K_{M}+K_{D}=\frac{E\left(1-d_{T}\right)}{3\left[1-2\nu +2\;d_{T}\left(1+\nu \right)\right]}>0,\;K_{C}\left(d_{C}\right)=K_{M}-K_{D}=\frac{E\left(1-d_{C}\right)}{3\left[1-2\nu +2\;d_{C}\left(1+\nu \right)\right]}>0,$$

the Helmholtz function’s values can be calculated as:

(15) $$H=\left\{\begin{array}{ll} \left(1-d\right)\left(\frac{3K_{T}}{2}p^{2}+G\;q^{2}\right) & \mathrm{for}\;p\geq 0,\\ \left(1-d\right)\left(\frac{3K_{C}}{2}p^{2}+G\;q^{2}\right) & \mathrm{for}\;p<0. \end{array}\right.$$

**Figure 1:**
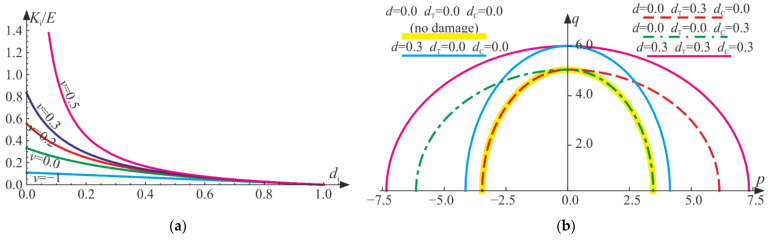
(**a**) Change of bulk moduli $K_{i}$ (i=T,C) due to increase in the respective damage parameters $d_{i}$ (i=T,C) with fixed Poisson’s ratio, according to Equation (14). (**b**) Contour lines H/E=const=10 of the considered Helmholtz potential, for ν=0.2 and various combinations of damage parameters’ values, according to Equation (15).

Equation (15) clearly shows that there is a difference between the stiffnesses for the extending strain states (p>0, compatible with tensile stress states, ξ>0) and the contracting strain states (p<0, compatible with compressive stress states, ξ<0), which will be further investigated. For p=0, the second derivative of the considered Helmholtz potential with respect to ε is not determined. $K_{T}\left(d_{T}\right)$ and $K_{C}\left(d_{C}\right)$ are bulk moduli for tension and compression, respectively. They are always both positive, irrespective of the level of degradation, which is shown in Figure 1a. The difference between $K_{T}$ and $K_{C}$ is imprinted on the shape of the contour lines of the free energy potential (see Figure 1b). Initially, for an isotropic undamaged material, the regarded contour is elliptic (yellow line), but due to the growth of $d_{T}$ or $d_{C}$ (decrease of the bulk moduli), it can stretch unequally in the p invariant direction (dashed red or green lines), respectively. When $d_{T}=d_{C}=0$ and d≠0, the contour stretches uniformly (blue line) with respect to the undamaged material. If all three parameters increase equally, the contour stretches to a new elliptic curve (pink line).

### 3.2. Dissipation Function

For concrete, and for quasibrittle materials in general, the form of the dissipation potential is rarely given in the literature. Since most of the elastic damage material models described in the literature are formulated by the damage condition dependent on the stress tensor, see [13,22,34,35], among others. However, the following general clues should be taken into consideration when designing the dissipation function’s form:

- The conjugate damage condition resembles yield conditions, and the damage surface in the Haigh–Westergaard coordinate system has curved meridian, open in the direction of compression and approximately triangular deviatoric cross section [40,41];
- Evolution laws connect damage parameters’ rates to some portions of the strain energy.

Adopting those suggestions, we propose the following dissipation potential:

(16) $$D\left(\dot{d},{\dot{d}}_{T},{\dot{d}}_{C};\sigma ,\varepsilon ,d,d_{T},d_{C}\right)=\kappa_{D}\left(\sigma ,\varepsilon ,d,d_{T},d_{C}\right)\sqrt{\frac{{\dot{d}}_{}^{2}}{Q_{}}+\frac{{\dot{d}}_{T}^{2}}{Q_{T}}+\frac{{\dot{d}}_{C}^{2}}{Q_{C}}},$$

where Q>0, $Q_{T}>0$, and $Q_{C}>0$ are material parameters, while $\kappa_{D}$ is:

(17) $$\kappa_{D}\left(\sigma ,\varepsilon ,d,d_{T},d_{C}\right)=\sqrt{Q\;h_{1}\left(\varepsilon ,d_{T},d_{C}\right)+Q_{T}h_{2}\left(\varepsilon ,d,d_{T}\right)+Q_{C}h_{3}\left(\varepsilon ,d,d_{C}\right)-F\left(\sigma \right)},\;h_{1}\left(\varepsilon ,d_{T},d_{C}\right)=\frac{3K_{M}\left(d_{T},d_{C}\right)}{2}p^{2}+\frac{3K_{D}\left(d_{T},d_{C}\right)}{2}\;p\sqrt{p^{2}}+G\;q^{2},\;h_{2}\left(\varepsilon ,d,d_{T}\right)=\frac{3\left(1-d\right)E\;p\left(p+\sqrt{p^{2}}\right)}{4{\left[1-2\nu +2\;d_{T}\left(1+\nu \right)\right]}^{2}},\;h_{3}\left(\varepsilon ,d,d_{C}\right)=\frac{3\left(1-d\right)E\;p\left(p-\sqrt{p^{2}}\right)}{4{\left[1-2\nu +2\;d_{C}\left(1+\nu \right)\right]}^{2}}.$$

$F\left(\sigma \right)$ is a convex function such that for any stress tensor, $F\left(\sigma \right)\leq 0$. Those conditions guarantee the required convexity and non-negativity of the considered potential. Dissipation expressed by Equation (16) is homogeneous of the degree 1 function of the rates $\dot{d}$, ${\dot{d}}_{T}$, and ${\dot{d}}_{C}$. Interpretation of the material parameters Q, $Q_{T}$, and $Q_{C}$ will be given in Section 4.

At this point, $F\left(\sigma \right)$ remains undefined to maintain the flexibility of the model. Function $F\left(\sigma \right)$ defines the target damage condition via the equation $F\left(\sigma \right)=0$. It is supposed to properly describe the real material behavior. We will give the details about it after some further derivations in Section 3.6.

The definition of functions in Equation (17) means that D is a function of both rates and nonrates (σ, ε, d, $d_{T}$, and $d_{C}$). The successive differentiations concern only $\dot{d}$, ${\dot{d}}_{T}$, and ${\dot{d}}_{C}$; the rest of the arguments are somewhat secondary. In the literature, this distinction is often denoted by a semicolon, that is, $D=D\left(\dot{d},{\dot{d}}_{T},{\dot{d}}_{C};\sigma ,\varepsilon ,d,d_{T},d_{C}\right)$.

### 3.3. Cauchy Stress and Relationships between Invariants of Stress and Strain Tensors

Using the first formula in Equation (1) for potential described by Equation (12), the following is derived:

(18) $$\begin{array}{l} \sigma =\frac{\partial \;H}{\partial \;\varepsilon }=\left(1-d\right)\left[\sqrt{3}\left(K_{M}p+K_{D}\sqrt{p^{2}}\right)\;I+2G\;e\right]=\\ \;\;\;\;=\left\{\begin{array}{ll} \left(1-d\right)\left(\sqrt{3}K_{T}p\;I+G\;e\right) & \mathrm{for}\;p\geq 0,\\ \left(1-d\right)\left(\sqrt{3}K_{C}p\;I+G\;e\right) & \mathrm{for}\;p<0. \end{array}\right. \end{array}$$

In an undamaged state (null values of damage parameters), Equation (18) reduces to the linear Hooke’s law of an isotropic material:

(19) $$K_{C}=K_{T}=\frac{E}{3\left(1-2\nu \right)}\;\Rightarrow \;\sigma =\frac{E}{3\left(1-2\nu \right)}\left(\mathrm{tr}\varepsilon \right)\;I+\frac{E}{1+\nu }e.$$

It is convenient to use the cylindrical invariants of the stress tensor σ, that is:

(20) $$\xi =\frac{1}{\sqrt{3}}\mathrm{tr}\sigma ,\;r=\sqrt{\mathrm{tr}s^{2}},\;\mathrm{and}\;\theta =\frac{1}{3}\arccos\frac{\sqrt{6}\;\mathrm{tr}s^{3}}{\sqrt{{\mathrm{tr}}^{3}s^{2}}},$$

where $s=\sigma -\frac{1}{3}\left(\mathrm{tr}\sigma \right)I$ is the stress tensor’s deviator. Splitting stress tensor described by Equation (18) into the hydrostatic and the deviatoric part, we obtain the following constitutive relationships between invariants specified by Equations (11) and (20):

(21) $$\xi =3\left(1-d\right)\left(K_{M}p+K_{D}\;\sqrt{p^{2}}\right)=\left\{\begin{array}{ll} 3\left(1-d\right)K_{T}p & \mathrm{for}\;p\geq 0,\\ 3\left(1-d\right)K_{C}p & \mathrm{for}\;p<0, \end{array}\right.\;s=2G\;\left(1-d\right)e=\;\left(1-d\right)\frac{E}{1+\nu }e,\;r=2G\;\left(1-d\right)q=\left(1-d\right)\frac{E}{1+\nu }q.$$

Equation (21) can be easily inverted to get the following useful relations:

(22) $$p=\frac{1}{3\left(1-d\right)}\left(\frac{K_{M}}{K_{M}^{2}-K_{D}^{2}}\xi -\frac{K_{D}}{K_{M}^{2}-K_{D}^{2}}\sqrt{\xi^{2}}\right)=\left\{\begin{array}{ll} \frac{\xi }{3\left(1-d\right)K_{T}} & \mathrm{for}\;\xi \geq 0,\\ \frac{\xi }{3\left(1-d\right)K_{C}} & \mathrm{for}\;\xi <0, \end{array}\right.\;e=\frac{1}{2G\;\left(1-d\right)}s=\frac{1+\nu }{E\;\left(1-d\right)}s,\;\;q=\frac{1}{2G\;\left(1-d\right)}r=\frac{1+\nu }{E\;\left(1-d\right)}r$$

and as a result:

(23) $$\varepsilon =\frac{1}{1-d}\left[\frac{K_{M}\xi -K_{D}\sqrt{\xi^{2}}}{3\sqrt{3}\left(K_{M}^{2}-K_{D}^{2}\right)}\;I+\frac{1}{2G}\;s\right]=\left\{\begin{array}{ll} \frac{1}{1-d}\left(\frac{\xi }{3\sqrt{3}K_{T}}\;I+\frac{1}{2G}\;s\right) & \mathrm{for}\;\xi \geq 0,\\ \frac{1}{1-d}\left(\frac{\xi }{3\sqrt{3}K_{C}}\;I+\frac{1}{2G}s\right) & \mathrm{for}\;\xi <0. \end{array}\right.$$

**Figure 2:**
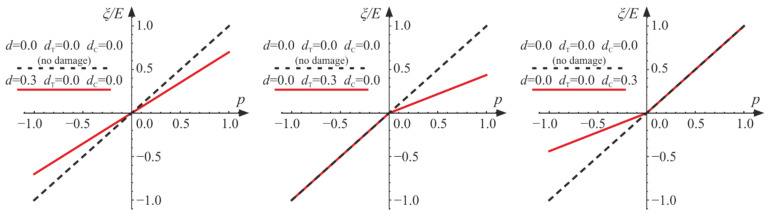
The influence of the damage parameters d, $d_{T}$, and $d_{C}$ on the relation between ξ and p for ν=0.2.

Derived dual constitutive relationships (Equations (18) and (23)) are homogeneous functions of degree 1 with respect to the strain and stress tensors, respectively. For fixed values of damage parameters, they define nonlinear constitutive relationships of an isotropic elastic material. The jump of stiffness regards the volumetric part of energy. It is clear that passing through p=0 (ξ=0), the slope of ξ−p curve changes discontinuously for a damaged material according to Equations (21) and (22) (see Figure 2). Graphical representations of the constitutive relations for the selected values of the damage parameters are associated with the contours of the Helmholtz energy given in Figure 1b. Due to an increase in the d parameter, the damaged material responds linearly with reduced bulk stiffness, so the parameter describes isotropic damage. If $d_{T}\neq 0$ or $d_{C}\neq 0$, a nonlinear response of material is described. An increase in $d_{T}$ results in a reduction of the bulk stiffness for tension, while an increase in $d_{C}$ describes a decrease in bulk stiffness for compression. So far, those responses are not limited by any damage (strength) criterion.

### 3.4. Generalized Stresses

Differentiating potential specified by Equation (12) according to the second formula in Equation (1), the following relations are obtained:

(24) $$\sigma_{d}=-\frac{\partial \;H}{\partial \;d}=\frac{H}{1-d}=\frac{3}{2}K_{M}p^{2}+\frac{3}{2}K_{D}\;p\sqrt{p^{2}}+G\;q^{2},\;\sigma_{dT}=-\frac{\partial \;H}{\partial \;d_{T}}=\frac{3\left(1-d\right)E\;p\left(p+\sqrt{p^{2}}\right)}{4{\left[1-2\nu +2\;d_{T}\left(1+\nu \right)\right]}^{2}}=\left\{\begin{array}{ll} \frac{3\left(1-d\right)E\;}{2{\left[1-2\nu +2\;d_{T}\left(1+\nu \right)\right]}^{2}}p^{2} & \mathrm{for}\;p\geq 0,\\ 0 & \mathrm{for}\;p<0, \end{array}\right.\;\sigma_{dC}=-\frac{\partial \;H}{\partial \;d_{C}}=\frac{3\left(1-d\right)E\;p\left(p-\sqrt{p^{2}}\right)}{4{\left[1-2\nu +2\;d_{C}\left(1+\nu \right)\right]}^{2}}=\left\{\begin{array}{ll} 0 & \mathrm{for}\;p\geq 0,\\ \frac{3\left(1-d\right)E\;}{2{\left[1-2\nu +2\;d_{C}\left(1+\nu \right)\right]}^{2}}p^{2} & \mathrm{for}\;p<0. \end{array}\right.$$

Let us notice that, in agreement with indications given in Section 3.2, the generalized stresses have units of energy. $\sigma_{d}$ is the Helmholtz energy divided by the factor 1−d, so it does not take into account isotropic damage, but it registers the degradation of material connected to the volumetric portion of the energy through moduli $K_{M}$ and $K_{D}$, which in turn depend on $d_{T}$ and $d_{C}$. The remaining two generalized stresses are associated with the scaled volumetric part $H_{K}$ of the considered Helmholtz energy in Equation (12), that is:

(25) $$\frac{\sigma_{dT}}{H_{K}}=\frac{\sigma_{dT}}{\frac{3}{2}\left(1-d\right)\left(K_{M}p^{2}+K_{D}p\sqrt{p^{2}}\right)}=\frac{3}{\left(1-d_{T}\right)\left[1-2\nu +2\;d_{T}\left(1+\nu \right)\right]}\;\mathrm{for}\;p>0\;,\frac{\sigma_{dC}}{H_{K}}=\frac{\sigma_{dC}}{\frac{3}{2}\left(1-d\right)\left(K_{M}p^{2}+K_{D}p\sqrt{p^{2}}\right)}=\frac{3}{\left(1-d_{C}\right)\left[1-2\nu +2\;d_{C}\left(1+\nu \right)\right]}\;\mathrm{for}\;p<0.$$

The generalized stresses may be expressed via the stress tensor’s invariants. Using Equation (22) in Equation (24) results in relationships:

(26) $$\sigma_{d}=\left\{\begin{array}{ll} \frac{1}{2{\left(1-d\right)}^{2}E}\left[\frac{1-2\nu +2\left(1+\nu \right)d_{T}}{1-d_{T}}\xi^{2}+\left(1+\nu \right)r^{2}\right] & \mathrm{for}\;\xi \geq 0,\\ \frac{1}{2{\left(1-d\right)}^{2}E}\left[\frac{1-2\nu +2\left(1+\nu \right)d_{C}}{1-d_{C}}\xi^{2}+\left(1+\nu \right)r^{2}\right] & \mathrm{for}\;\xi <0, \end{array}\right.\;\sigma_{dT}=\left\{\begin{array}{ll} \frac{3\;\xi^{2}\;}{2\left(1-d\right){\left(1-d_{T}\right)}^{2}E} & \mathrm{for}\;\xi \geq 0,\\ 0 & \mathrm{for}\;\xi <0, \end{array}\right.\sigma_{dC}=\left\{\begin{array}{ll} 0 & \mathrm{for}\;\xi \geq 0,\\ \frac{3\;\xi^{2}\;}{2\left(1-d\right){\left(1-d_{C}\right)}^{2}E} & \mathrm{for}\;\xi <0. \end{array}\right.$$

### 3.5. Dissipative Stresses, Damage Condition, and Evolution Laws

The dissipative stresses described by Equation (2) are calculated as the following derivatives of the proposed dissipation in Equation (16):

(27) $${\bar{\sigma }}_{d}=\frac{\partial \;D}{\partial \;\dot{d}}=\frac{\kappa_{D}^{2}{\dot{d}}_{C}^{}}{Q\;D},\;{\bar{\sigma }}_{dT}=\frac{\partial \;D}{\partial \;{\dot{d}}_{T}}=\frac{\kappa_{D}^{2}{\dot{d}}_{T}^{}}{Q_{T}D},\;{\bar{\sigma }}_{dC}=\frac{\partial \;D}{\partial \;{\dot{d}}_{C}}=\frac{\kappa_{D}^{2}{\dot{d}}_{C}^{}}{Q_{C}D}.$$

In turn, the damage condition (Equation (6)) becomes:

(28) $$\bar{F}\left({\bar{\sigma }}_{d},{\bar{\sigma }}_{dT},{\bar{\sigma }}_{dC}\right)=Q\;{\bar{\sigma }}_{d}^{2}+Q_{T}{\bar{\sigma }}_{dT}^{2}+Q_{C}{\bar{\sigma }}_{dC}^{2}-\kappa_{D}^{2}=0,$$

and the evolution laws (Equation (8)) are:

(29) $$\dot{d}=\lambda \frac{\partial \;\bar{F}}{{\bar{\sigma }}_{d}}=2\lambda Q\;{\bar{\sigma }}_{d}^{},\;{\dot{d}}_{C}=\lambda \frac{\partial \;\bar{F}}{{\bar{\sigma }}_{dC}}=2\lambda Q_{C}{\bar{\sigma }}_{dC}^{},\;{\dot{d}}_{T}=\lambda \frac{\partial ;\bar{F}}{{\bar{\sigma }}_{dT}}=2\lambda Q_{T}{\bar{\sigma }}_{dT}^{}.$$

Equations (28) and (29) are complemented by the consistency condition (Equation (7)).

### 3.6. Orthogonality Principle, Damage Condition, and Evolution Laws Expressed via Cauchy Stress

In the considered case, the orthogonality principle (Equation (9)) establishes three equalities:

(30) $${\bar{\sigma }}_{d}=\sigma_{d},\;{\bar{\sigma }}_{dT}=\sigma_{dT},\;\mathrm{and}\;{\bar{\sigma }}_{dC}=\sigma_{dC},$$

which bind spaces of the generalized and the dissipative stresses. Now, using the definition of $\kappa_{D}^{}$ according to Equation (17) and of the generalized stresses (Equation (26)), Equation (28) can be written as:

(31) $$F\left(\sigma \right)=0$$

and the evolution laws (Equation (29)) are in the form:

(32) $$\dot{d}=2\lambda Q\;\sigma_{d}^{},\;{\dot{d}}_{C}=2\lambda Q_{C}\sigma_{dC}^{},\;{\dot{d}}_{T}=2\lambda Q_{T}\sigma_{dT}^{}.$$

Consistency condition expressed via Cauchy stress tensor reads:

(33) $$\dot{F}\left(\sigma \right)=0.$$

**Figure 3:**
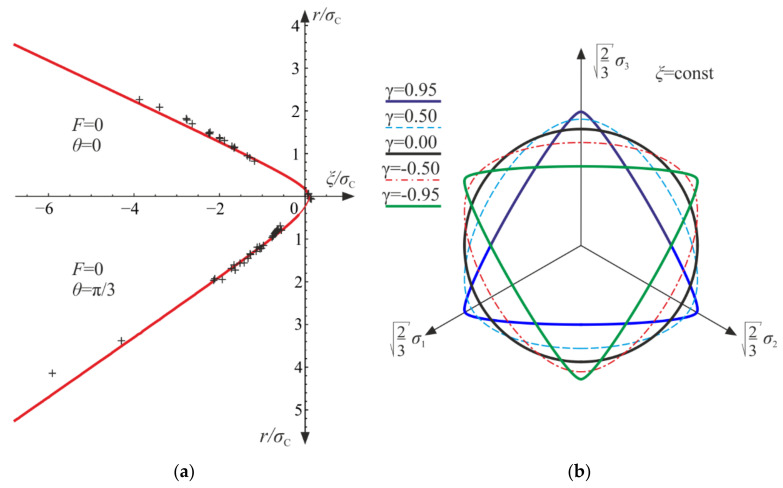
The shape of the considered damage surface described by Equation (34) in the Haigh–Westergaard coordinate system: (**a**) tensile and compressive meridians compared with experimental data [25,26,32] for $\sigma_{T}=0.1\;\sigma_{C}$, $\sigma_{BC}=1.16\;\sigma_{C}$, $\sigma_{TC}=1.26\;\sigma_{C}$, and η=4.91, resulting in $c=0.805\;\sigma_{C}$, $b=0.515\;\sigma_{C}$, B=1.41, and γ=−0.834 (**b**) deviatoric cross sections of F=0 for various γ. $\sigma_{1}$, $\sigma_{2}$, and $\sigma_{3}$ are unordered principal values of the stress tensor.

In order to conduct further derivations, we need to specify the function $F\left(\sigma \right)$, which properly captures a real material behavior. For concrete and rocks, we may assume that:

(34) $$F\left(\xi ,r,\theta \right)=3\;C\left(\xi \right)r^{2}-2\sqrt{\left(1-\gamma^{2}\right){\left(C{\left(\xi \right)}^{2}-r^{2}\right)}^{3}}-\;\left(2-\gamma^{2}\right)C{\left(\xi \right)}^{3}-2\;\gamma \;r^{3}\cos3\theta =0,\;\mathrm{with}\;C\left(\xi \right)=\sqrt{{\left(\frac{c-\xi }{B}\right)}^{2}-b^{2}},$$

where c>0, b>0, B>0, and −1<γ<1 are material parameters. Function F is convex for any ξ, r, and θ. The damage surface F=0 possesses the desired features mentioned in Section 3.2; see the cross sections of the damage surface in Figure 3, where the shapes of tensile (θ=0) and compressive (θ=π/3) meridians are compared with experimental data, and possible shapes of the deviatoric cross sections are shown.

Finding the four parameters c, b, B, and γ involved in the damage condition requires knowing the damage limits (strengths) for four independent tests. For concrete, the basic experiment is the uniaxial compression. Additionally, the uniaxial tension, biaxial equilateral compression, and triaxial compression tests are representative and well grounded in the literature. Let $\sigma_{T}$, $\sigma_{C}$, $\sigma_{BC}$, and $\eta \;\sigma_{TC}$ with η>1 be the damage limits for the uniaxial tension, uniaxial compression, biaxial equilateral compression, and triaxial compression test with $\sigma \to \mathrm{diag}[-\eta \sigma_{TC},-\sigma_{TC},-\sigma_{TC}]$ (the stress tensor is represented by the shown diagonal matrix), respectively. The tests are located on either the tensile meridian (θ=0) or the compressive meridian (θ=π/3), and for those cases, damage condition expressed by Equation (34) reduces to quite simple expressions (for particulars, see [40]). This useful feature allows for solving the set of four equations—$F\left(\xi_{T},r_{T},0\right)=0$, $F\left(\xi_{BC},r_{BC},0\right)=0$, $F\left(\xi_{C},r_{C},\pi /3\right)=0$, and $F\left(\xi_{TC},r_{TC},\pi /3\right)=0$—for the unknown parameters c, b, B, and γ. The values of the cylindrical invariants, defined by Equation (20), for the specified cases are:

(35) $$\xi_{T}=\frac{\sigma_{T}}{\sqrt{3}},\;\xi_{C}=-\frac{\sigma_{C}}{\sqrt{3}},\;\xi_{BC}=-\frac{2\sigma_{BC}}{\sqrt{3}},\;\xi_{C}=-\frac{\left(\eta +2\right)\sigma_{C}}{\sqrt{3}},\;r_{T}=\sqrt{\frac{2}{3}}\;\sigma_{T},\;r_{C}=\sqrt{\frac{2}{3}}\;\sigma_{C},\;r_{BC}=\sqrt{\frac{2}{3}}\;\sigma_{BC},\;r_{TC}=\sqrt{\frac{2}{3}}\;\left(\eta +1\right)\sigma_{TC}.$$

Introducing notations:

(36) $$\tilde{a}=\frac{\xi_{BC}r_{T}^{2}-\xi_{T}r_{BC}^{2}}{r_{BC}^{2}-r_{T}^{2}},\;\;\tilde{b}=\frac{\xi_{TC}r_{C}^{2}-\xi_{C}r_{TC}^{2}}{r_{TC}^{2}-r_{C}^{2}},\;\;\tilde{c}=\frac{\xi_{BC}^{2}r_{T}^{2}-\xi_{T}^{2}r_{BC}^{2}}{r_{BC}^{2}-r_{T}^{2}},\;\;\tilde{d}=\frac{\xi_{TC}^{2}r_{C}^{2}-\xi_{C}^{2}r_{TC}^{2}}{r_{TC}^{2}-r_{C}^{2}}$$

the following formulae for parameters involved in the damage condition are derived:

(37) $$c=\frac{\tilde{c}-\tilde{d}}{2(\tilde{a}-\tilde{b})},\;\;t=\frac{r_{C}}{r_{T}}\sqrt{\frac{\left(\tilde{b}-\tilde{a}\right)\xi_{T}^{2}+\left(\tilde{c}-\tilde{d}\right)\xi_{T}-\tilde{a}\;\tilde{d}+\tilde{b}\;\tilde{c}}{\left(\tilde{b}-\tilde{a}\right)\xi_{C}^{2}+\left(\tilde{c}-\tilde{d}\right)\xi_{C}-\tilde{a}\;\tilde{d}+\tilde{b}\;\tilde{c}}},\;\;\gamma =\frac{3\sqrt{3}\;t\;(1-t)}{2\sqrt{{\left(t^{2}-t+1\right)}^{3}}},\;b=\frac{r_{T}}{2\cos\left(\frac{1}{3}\arccos\gamma \right)}\sqrt{\frac{{\left(\tilde{c}-\tilde{d}\right)}^{2}+4\left(\tilde{a}\;\tilde{d}-\tilde{b}\;\tilde{c}\right)\left(\tilde{b}-\tilde{a}\right)}{\left(\tilde{b}-\tilde{a}\right)\left[\left(\tilde{b}-\tilde{a}\right)\xi_{T}^{2}+\left(\tilde{c}-\tilde{d}\right)\xi_{T}-\tilde{a}\;\tilde{d}+\tilde{b}\;\tilde{c}\right]}},\;B=\frac{1}{b}\sqrt{\frac{1}{4}{\left(\frac{\tilde{c}-\tilde{d}}{\tilde{b}-\tilde{a}}\right)}^{2}+\frac{\tilde{a}\;\tilde{d}-\tilde{b}\;\tilde{c}}{\tilde{b}-\tilde{a}}}.$$

A detailed description of the function’s F properties with suitable graphs and particulars of the calibration procedure can be found in [40].

Obtaining the closed formulae for material parameters is useful, especially with regard to hardening. Hardening can be taken into account if we make the limits (strengths) $\sigma_{T}$, $\sigma_{C}$, $\sigma_{BC}$, and $\eta \;\sigma_{TC}$ dependent on the hardening variables, which can be the damage parameters, meaning that the limits, instead of being constant, are assumed to be the functions $\sigma_{T}=\sigma_{T}\left(d,d_{T},d_{C}\right)$, and so forth. Then Equation (37) allow for directly finding c, b, B, and γ as functions of the damage parameters. In this way, the damage function F becomes dependent on σ, d, $d_{T}$, and $d_{C}$. The subsequent derivations will be based on the assumption that hardening is dependent on those parameters. If any of the parameters c, b, B, and γ were sought by curve fitting, the procedure would be quite complicated. The existence of the closed calibration formulae simplifies the issue. Still, the damage condition is fairly complex and quite general.

### 3.7. Tensor of Tangent Stiffness of Elastic Damaged Material

Let us calculate the increment of $\sigma \left(\varepsilon ,d,d_{T},d_{C}\right)$ based on Equation (18):

(38) $$\begin{array}{c} \dot{\sigma }=\frac{\partial \;\sigma }{\partial \;\varepsilon }\cdot \dot{\varepsilon }+\frac{\partial \;\sigma }{\partial \;d}\dot{d}+\frac{\partial \;\sigma }{\partial \;d_{T}}{\dot{d}}_{T}+\frac{\partial \;\sigma }{\partial \;d_{C}}{\dot{d}}_{C}=\\ =\left(1-d\right)\left[\left(K_{M}+K_{D}\frac{p}{\sqrt{p^{2}}}-\frac{2}{3}G\right)\;I⊗I+2G\;1\right]\cdot \dot{\varepsilon }+\\ -\dot{d}\left[\sqrt{3}\left(K_{M}p+K_{D}\sqrt{p^{2}}\right)\;I+2G\;e\right]+\sqrt{3}\left(1-d\right)\left\{{\dot{d}}_{T}g_{T}\left(p+\sqrt{p^{2}}\right)+{\dot{d}}_{C}g_{C}\left(p-\sqrt{p^{2}}\right)\right\}I, \end{array}$$

where · denotes the full contraction of tensors, ⊗ denotes the tensor product, $1\;=\frac{1}{2}\left(\delta_{ik}\delta_{jl}+\delta_{il}\delta_{jk}\right)b_{i}⊗b_{j}⊗b_{k}⊗b_{l}$ is for i,j,k,l=1,2,3 using the summation convention, $\left\{b_{1},b_{2},b_{3}\right\}$ is an orthonormal basis, and $\delta_{ik}$ is the Kronecker delta, and:

(39) $$g_{T}\left(d_{T}\right)=\frac{\partial K_{M}}{\partial d_{T}}=\frac{\partial K_{D}}{\partial d_{T}}=-\frac{E}{2{\left(1-2\nu +2d_{T}(1+\nu )\right)}^{2}},\;g_{C}\left(d_{C}\right)=\frac{\partial K_{M}}{\partial d_{C}}=-\frac{\partial K_{D}}{\partial d_{C}}=-\frac{E}{2{\left(1-2\nu +2d_{C}(1+\nu )\right)}^{2}}.$$

Now, we use the consistency condition described by Equation (33):

(40) $$\dot{F}=\left(\frac{\partial F}{\partial \xi }\frac{\partial \xi }{\partial \sigma }+\frac{\partial F}{\partial r}\frac{\partial r}{\partial \sigma }+\frac{\partial F}{\partial \cos3\theta }\frac{\partial \cos3\theta }{\partial \sigma }\right)\cdot \dot{\sigma }+\frac{\partial F}{\partial d}\dot{d}+\frac{\partial F}{\partial d_{T}}{\dot{d}}_{T}+\frac{\partial F}{\partial d_{C}}{\dot{d}}_{C}=0.$$

Three last components appear as a result of hardening (or softening) dependent on the current values of damage parameters. Introducing:

(41) $$f_{1}=\frac{\partial F}{\partial C}=3\left[r^{2}-C^{2}(2-\gamma^{2})-2C\sqrt{\left(1-\gamma^{2})(C^{2}-r^{2}\right)}\right],\;C_{\xi }=\frac{\partial C}{\partial \xi }=-\frac{c-\xi }{B\sqrt{{\left(c-\xi \right)}^{2}-b^{2}B^{2}}},\;f_{2}=\frac{\partial F}{\partial r}=6\;r\left(C+\sqrt{\left(1-\gamma^{2}\right)\left(C^{2}-r^{2}\right)}-\gamma \;r\;\cos3\theta \right),\;f_{3}=\frac{\partial F}{\partial \cos3\theta }=-2\;\gamma \;r^{3},\;g_{1}=\frac{\partial \xi }{\partial \sigma }=\frac{1}{\sqrt{3}}Ι,\;g_{2}=\frac{\partial r}{\partial \sigma }=\frac{1}{r}s,\;g_{3}=\frac{\partial \cos3\theta }{\partial \sigma }=\frac{3\sqrt{6}}{r^{3}}\left(s^{2}-\frac{r\;\cos3\theta }{\sqrt{6}}s-\frac{r^{2}}{3}I\right),\;K_{0}=K_{M}+K_{D}\frac{p}{\sqrt{p^{2}}}\;\mathrm{and}\;S_{0}=Q_{T}\sigma_{dT}^{}g_{T}\left(p+\sqrt{p^{2}}\right)+Q_{C}\sigma_{dC}^{}g_{C}\left(p-\sqrt{p^{2}}\right)$$

and taking into account the evolution laws (Equation (32)), the consistency condition (Equation (40)) and the stress increment (Equation (38)) become:

(42) $$\dot{F}=\left(f_{1}C_{\xi }g_{1}+f_{2}\;g_{2}+f_{3}\;g_{2}\right)\cdot \dot{\sigma }+2\lambda \left(\frac{\partial F}{\partial d}Q\sigma_{d}^{}+\frac{\partial F}{\partial d_{T}}Q_{T}\sigma_{dT}^{}+\frac{\partial F}{\partial d_{C}}Q_{C}\sigma_{dC}^{}\right)=0,$$

(43) $$\dot{\sigma }=\left(1-d\right)\left[\left(K_{0}-\frac{2}{3}G\right)I⊗I+2G\;1\right]\cdot \dot{\varepsilon }-2\lambda \left[Q\sigma_{d}^{}\left(\sqrt{3}K_{0}p\;I+2G\;e\right)+\sqrt{3}\left(1-d\right)S_{0}I\right].$$

Using the above formula for the stress increment, we solve Equation (42) to obtain the damage multiplier:

(44) $$\lambda =\frac{1}{2R_{0}}\left(1-d\right)\left[\sqrt{3}f_{1}C_{\xi }K_{0}I+2G\left(f_{2}g_{2}+f_{3}g_{3}\right)\right]\cdot \dot{\varepsilon },\;\mathrm{where}\;\begin{array}{c} R_{0}=Q\sigma_{d}^{}\left(3f_{1}C_{\xi }K_{0}p+\;\frac{f_{2}r}{\;1-d}\right)-\sqrt{3}\left(1-d\right)f_{1}C_{\xi }S_{0}+\\ -\left(Q\sigma_{d}^{}\frac{\partial F}{\partial d}+Q_{T}\sigma_{dT}^{}\frac{\partial F}{\partial d_{T}}\;+Q_{C}\sigma_{dC}^{}\frac{\partial F}{\partial d_{C}}\right). \end{array}$$

The final step in the derivation of the incremental form of the constitutive relationship is to apply Equation (44) in Equation (43), which results in:

(45) $$\dot{\sigma }=C^{\tan}\cdot \dot{\varepsilon },$$

where $C^{\tan}$ is the sought tensor of the tangent stiffness of the considered elastic damaged material:

(46) $$C^{\tan}\;=\left(1-d\right)\left(C_{0}-C_{D}\right)$$

with:

(47) $$C_{0}=\left(K_{0}-\frac{2}{3}G\right)\;I⊗I+2G\;1,\;C_{D}=\frac{1}{R_{0}}c_{1}⊗c_{2},\;c_{1}=\left(\sqrt{3}Q_{}\sigma_{d}K_{0}p-\left(1-d\right)S_{0}\right)I+2G\;Q\sigma_{d}\;e,\;c_{2}=\sqrt{3}f_{1}C_{\xi }K_{0}I+2G\left(f_{2}g_{2}+f_{3}g_{3}\right).$$

Let us emphasize that all the proper symmetries, that is, $C_{ijkl}^{\tan}=\;C_{klij}^{\tan}=C_{jikl}^{\tan}=C_{ijlk}^{\tan}$, occur. The tensor is determined for $p\in R\backslash \left\{0\right\}$ ($\xi \in R\backslash \left\{0\right\}$).

We can also derive the fourth-order tensor of the secant stiffness $C^{\sec}$:

(48) $$\sigma =C^{\sec}\cdot \varepsilon ,$$

merely by rearranging Equation (18). The following is obtained:

(49) $$C^{\sec}=\left(1-d\right)\left[\left(K_{0}-\frac{2}{3}G\;\right)I⊗I+2G\;1\right]=\left(1-d\right)C_{0}.$$

## 4. Model’s Predictions for Uniaxial Stress States and Pure Shear

Below we present the numerical results for the uniaxial tension, compression, and cyclic tension–compression and pure shear omitting hardening. The aim of the calculations is to show the basic features of the model, to assess the quality of results rather than their quantity. We seek for physical (experimental) interpretation of the damage parameters d, $d_{T}$, and $d_{C}$ and investigate the influence of the parameters Q, $Q_{T}$, and $Q_{C}$ on the model predictions. Including hardening is quite simple, but it blurs the equations, so for the sake of clarity, it is left out in most cases.

For the chosen tests, the equations are simple enough to use basic solvers available in Wolfram Mathematica; there is no necessity for creating a special finite element code, although for more complex problems, it would be inevitable.

### 4.1. Uniaxial Tension

For the uniaxial tension, the strain and the stress states are:

(50) $$\varepsilon =\varepsilon_{11}b_{1}⊗b_{1}+\varepsilon_{22}b_{2}⊗b_{2}+\varepsilon_{33}b_{3}⊗b_{3},\;\;\sigma =\sigma_{11}\;b_{1}⊗b_{1},\;\mathrm{and}\;\sigma_{11}\geq 0.$$

As invariants defined by Equation (20) for this case are:

(51) $$\xi =\frac{1}{\sqrt{3}}\sigma_{11},\;r=\sqrt{\frac{2}{3}}\;\sigma_{11},\;\theta =0,$$

the invariants of ε can be found using constitutive relations for the volumetric and distortional parts according to Equation (22), become:

(52) $$p=\frac{1-2\nu +2d_{T}\left(1+\nu \right)}{\sqrt{3}\left(1-d\right)\left(1-d_{T}\right)E}\sigma_{11}\;\mathrm{and}\;q=\sqrt{\frac{2}{3}}\frac{1+\nu }{\;E\;\left(1-d\right)}\sigma_{11},$$

so the strain tensor is:

(53) $$\varepsilon =\frac{1}{\left(1-d\right)E}\left[\frac{1-2\nu +2d_{T}\left(1+\nu \right)}{3\left(1-d_{T}\right)}\sigma_{11}\;I+\left(1+\nu \right)\;s\right]\to \left[\begin{array}{ccc} \varepsilon_{11} & 0 & 0\\ 0 & \varepsilon_{22} & 0\\ 0 & 0 & \varepsilon_{33} \end{array}\right].$$

The above constitutive equation is equivalent to the following relation between the stress and strain components:

(54) $$\varepsilon_{11}=\frac{\sigma_{11}}{\left(1-d\right)\left(1-d_{T}\right)E},\;\varepsilon_{22}=\varepsilon_{33}=-\left[\nu -d_{T}\left(1+\nu \right)\right]\varepsilon_{11},$$

which, for an undamaged state of material, reduce to the well-known Hooke’s law:

(55) $$\varepsilon_{11}=\frac{\sigma_{11}}{E},\;\varepsilon_{22}=\varepsilon_{33}=-\nu \;\varepsilon_{11}.$$

Let $\varepsilon_{11}$ be dependent on time t and prescribed by the formula:

(56) $$\varepsilon_{11}\left(t\right)=a\;t,\;a>0.$$

Then using Equation (54), we obtain:

(57) $$\sigma_{11}\left(t\right)=\left[1-d\left(t\right)\right]\left[1-d_{T}\left(t\right)\right]E\;a\;t,\;\varepsilon_{22}\left(t\right)=\varepsilon_{33}\left(t\right)=-\left[\nu -d_{T}\left(t\right)\left(1+\nu \right)\right]a\;t.$$

The loading process starting from a natural state, that is, σ=0, ε=0, d=0, $d_{T}=0$, and $d_{C}=0$, is purely elastic until the damage limit for the uniaxial tension $\sigma_{T}$ is attained. The following equations remain true:

(58) $$\sigma_{11}\left(t\right)=Eat,\;\varepsilon_{22}\left(t\right)=\varepsilon_{33}\left(t\right)=-\nu \;a\;t,\;d\left(t\right)=0,\;d_{T}\left(t\right)=0,\;d_{C}\left(t\right)=0$$

for $t<t_{0}=\sigma_{T}/\left(E\;a\right)$. Further increase in strain according to the program prescribed by Equation (56) leads to a development of damage. The damage condition defined by Equation (34) reduces to:

(59) $$\sigma_{11}-\sigma_{T}=0.$$

The generalized stresses defined by Equation (24) become:

(60) $$\sigma_{d}^{}=\frac{E\left(1-d_{T}\right)}{2}\varepsilon_{11}^{2},\;\sigma_{dT}^{}=\frac{E\left(1-d\right)}{2}\varepsilon_{11}^{2},\;\sigma_{dC}^{}=0.$$

In turn, the evolution laws (Equation (32)) take the form:

(61) $$\dot{d}=\lambda QE\left(1-d_{T}\right)\varepsilon_{11}^{2},\;{\dot{d}}_{T}=\lambda Q_{T}E\left(1-d\right)\varepsilon_{11}^{2},\;{\dot{d}}_{C}=0.$$

As a result, the compressive damage parameter stays null throughout the process, that is, $d_{C}\left(t\right)=0$, and for $t\geq t_{0}$, the other quantities must meet the following conditions:

(62) $$\left\{\begin{array}{c} \left(1-d\left(t\right)\right)\left(1-d_{T}\left(t\right)\right)=\frac{t_{0}}{t},\;\;\;\;\;\\ \dot{d}\left(t\right)\left(1-d\left(t\right)\right)Q_{T}={\dot{d}}_{T}\left(t\right)\left(1-d_{T}\left(t\right)\right)Q,\\ d_{T}\left(t_{0}\right)=0,\;\;d\left(t_{0}\right)=0.\;\;\;\;\;\; \end{array}\right.$$

The above differential equations are solved numerically using Wolfram Mathematica for the following values of material constants: E=20 GPa, ν=0.2, $\sigma_{T}=2\;\mathrm{MPa}$, and $a=0.0001\;s^{-1}$, Q=1, $Q_{T}=2$. For these data, the damage limit is reached at $t_{0}=1\;s$.

**Figure 4:**
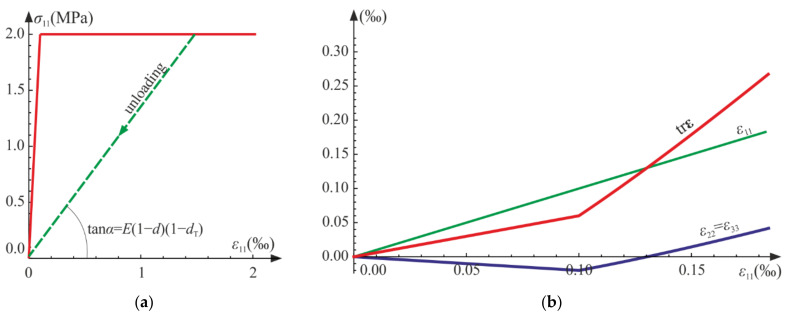
Uniaxial tension: (**a**) axial strain versus stress curve; (**b**) strain components and the trace of ε versus axial strain $\varepsilon_{11}$.

**Figure 5:**
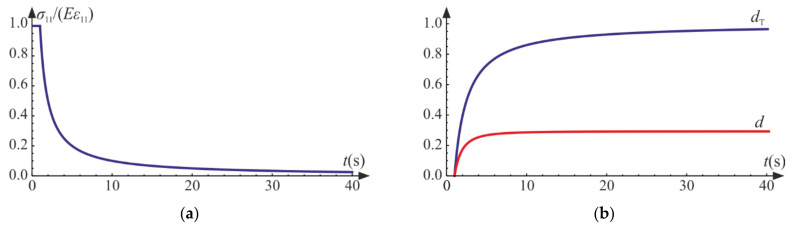
Uniaxial tension: (**a**) scaled secant stiffness versus time; (**b**) damage parameters versus time.

Initially, the material is elastic with stiffness equal to the initial Young’s modulus E. After reaching the damage limit, the stress becomes constant due to the assumed lack of hardening, as presented in Figure 4a. The secant stiffness becomes $\left(1-d\right)\left(1-d_{T}\right)E$. The degradation of the initial stiffness (see Figure 5a) mirrors the decrease in the current slope of a hypothetic unloading curve, $\sigma_{11}-\varepsilon_{11}$. As shown in Figure 4b, the lateral strain components decrease to the limit point, but after the degradation process origins, they start to increase. As a result, the trace of the strain tensor, that is, the relative change of volume, increases approximately piecewise linearly (for $\sigma_{11}=\sigma_{T}$, it is nonlinear). A more dynamic growth of the volumetric strain is observed as the degradation enters. As d→1 or $d_{T}\to 1$ (Figure 5b), the ability of material to carry any loading vanishes, and total failure occurs. It is reasonable to establish arbitrary limit values of the damage parameters $d_{0}<1$ and $d_{T0}<1$, marking a partial but severe-enough level of degradation.

Knowing the experimental curves of the axial and lateral strains, we can easily find a function of the tensile damage parameter based on Equation (54):

(63) $$d_{T}=-\frac{1}{1+\nu }\left(\frac{\varepsilon_{22}}{\varepsilon_{11}}+\nu \right).$$

Thus, the progress of $d_{T}$ can be found directly in experimental results. In the uniaxial tension, factor $\left(1+\nu \right)d_{T}$ lowers the initial Poisson’s ratio ν.

**Figure 6:**
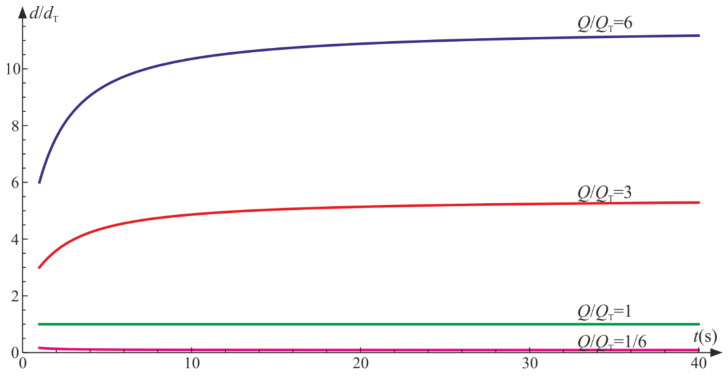
Uniaxial tension: ratio of damage parameters versus $Q/Q_{T}$.

Figure 6 depicts the evolution of damage parameters for various ratios, $Q/Q_{T}$. We set Q=1 and control the model prediction by changing $Q_{T}$. It is apparent that by increasing $Q/Q_{T}$, the isotropic damage parameter prevails. On the contrary, a decrease in the quotient lets the tensile damage parameter be the leading function in the material degradation process. For $Q/Q_{T}<1$, the degradation due to the change of volume is bigger than the isotropic degradation ($d/d_{T}<1$), and vice versa for $Q/Q_{T}>1$, we have $d/d_{T}>1$.

### 4.2. Uniaxial Compression

The strain and stress tensors for the uniaxial compression are the same as for the uniaxial tension excluding signs of the components, that is:

(64) $$\varepsilon =\varepsilon_{11}b_{1}⊗b_{1}+\varepsilon_{22}b_{2}⊗b_{2}+\varepsilon_{33}b_{3}⊗b_{3},\;\;\sigma =\sigma_{11}\;b_{1}⊗b_{1},\;\mathrm{and}\;\sigma_{11}\leq 0.$$

As a result, the cylindrical invariants defined by Equation (20) become:

(65) $$\xi =\frac{1}{\sqrt{3}}\sigma_{11},\;r=-\sqrt{\frac{2}{3}}\;\sigma_{11},\;\theta =\pi /3.$$

Next, using the constitutive relationship between invariants (Equation (22)), we obtain:

(66) $$p=\frac{1-2\nu +2d_{C}\left(1+\nu \right)}{\sqrt{3}\left(1-d\right)\left(1-d_{C}\right)E}\sigma_{11},\;q=-\frac{1}{\sqrt{6}\;G\;\left(1-d\right)}\sigma_{11}$$

and:

(67) $$\varepsilon =\frac{1}{\left(1-d\right)E}\left[\frac{1-2\nu +2d_{C}\left(1+\nu \right)}{3\left(1-d_{C}\right)}\sigma_{11}\;I+\left(1+\nu \right)\;s\right]\to \left[\begin{array}{ccc} \varepsilon_{11} & 0 & 0\\ 0 & \varepsilon_{22} & 0\\ 0 & 0 & \varepsilon_{33} \end{array}\right].$$

The above constitutive equation is equivalent to the following relationships between the stress and strain components:

(68) $$\varepsilon_{11}=\frac{\sigma_{11}}{\left(1-d\right)\left(1-d_{C}\right)E},\;\varepsilon_{22}=\varepsilon_{33}=-\left[\nu -d_{C}\left(1+\nu \right)\right]\varepsilon_{11}.$$

As in Section 4.1, Equation (68) for the undamaged material reduce to Hooke’s law (Equation (55)).

Again, we assume that $\varepsilon_{11}$ is dependent on time as follows:

(69) $$\varepsilon_{11}\left(t\right)=-a\;t,\;a>0.$$

Then using Equation (68), we obtain:

(70) $$\sigma_{11}\left(t\right)=-\left[1-d\left(t\right)\right]\left[1-d_{C}\left(t\right)\right]E\;a\;t,\;\varepsilon_{22}\left(t\right)=\varepsilon_{33}\left(t\right)=\left[\nu -d_{C}\left(t\right)\left(1+\nu \right)\right]a\;t.$$

For the elastic range, that is, as long as the axial stress does not reach the damage limit for the uniaxial compression $\sigma_{C}$ or $t<t_{0}=\sigma_{C}/\left(E\;a\right)$, the governing equations are:

(71) $$\sigma_{11}\left(t\right)=-Ea\;t,\;\varepsilon_{22}\left(t\right)=\varepsilon_{33}\left(t\right)=\nu \;a\;t,\;d\left(t\right)=0,\;d_{T}\left(t\right)=0,\;d_{C}\left(t\right)=0.$$

The subsequent loading leads to deterioration of material. The damage condition expressed by Equation (34) takes the form:

(72) $$\sigma_{11}+\sigma_{C}=0.$$

The generalized stresses (Equation (24)) are:

(73) $$\sigma_{d}^{}=\frac{E\left(1-d_{C}\right)}{2}\varepsilon_{11}^{2},\;\sigma_{dT}^{}=0,\;\sigma_{dC}^{}=\frac{E\left(1-d\right)}{2}\varepsilon_{11}^{2},$$

so the evolution laws (Equation (32)) reduce to:

(74) $$\dot{d}=\lambda QE\left(1-d_{C}\right)\varepsilon_{11}^{2},\;{\dot{d}}_{T}=0,\;{\dot{d}}_{C}=\lambda Q_{C}E\left(1-d\right)\varepsilon_{11}^{2}.$$

Contrary to the uniaxial tension, $d_{T}\left(t\right)=0$, while $d_{C}$ increases. For $t\geq t_{0}$, the following system governs the process:

(75) $$\left\{\begin{array}{c} \left(1-d\left(t\right)\right)\left(1-d_{C}\left(t\right)\right)=\frac{t_{0}}{t},\;\;\;\;\;\\ \dot{d}\left(t\right)\left(1-d\left(t\right)\right)Q_{C}={\dot{d}}_{C}\left(t\right)\left(1-d_{C}\left(t\right)\right)Q,\\ d_{C}\left(t_{0}\right)=0,\;\;d\left(t_{0}\right)=0.\;\;\;\;\;\; \end{array}\right.$$

The above set of equations is solved numerically using the NDSolve procedure of Wolfram Mathematica for E=20 GPa, ν=0.2, $\sigma_{C}=20\;\mathrm{MPa}$, and $a=0.0001\;s^{-1}$, Q=1 and $Q_{C}=5$. The damage limit is attained at $t_{0}=10\;s$.

**Figure 7:**
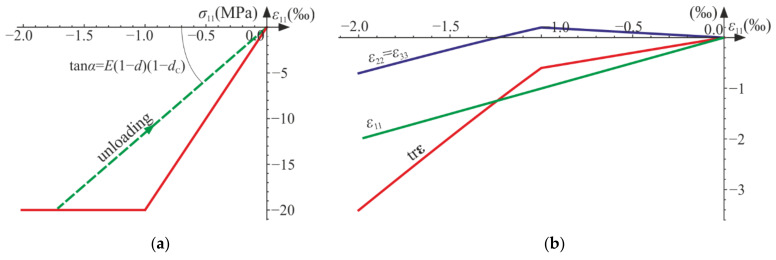
Uniaxial compression: (**a**) axial strain versus stress curve; (**b**) strain components and the trace of ε versus axial strain $\varepsilon_{11}$.

**Figure 8:**
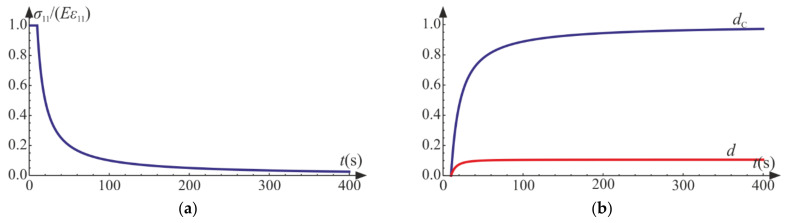
Uniaxial compression: (**a**) scaled secant stiffness versus time; (**b**) damage parameters versus time.

As shown in Figure 7 and Figure 8, the characteristics of the obtained function are similar to that for the uniaxial tension. When the degradation process is active, the secant stiffness is lower compared with the initial value and is scaled by factor $\left(1-d\right)\left(1-d_{T}\right)$. As damage develops factor $\left(1-d\right)\left(1-d_{C}\right)$ tends to zero, which leads to complete failure of material.

Analogous to Equation (63), the compressive damage parameter can be determined by knowing the ratio of the strain tensor components (the generalized Poisson’s ratio function) and the initial Poisson’s ratio according to:

(76) $$d_{C}=-\frac{1}{1+\nu }\left(\frac{\varepsilon_{22}}{\varepsilon_{11}}+\nu \right).$$

This time factor $\left(1+\nu \right)d_{C}$ is subtracted from ν to obtain the current ratio of strains.

**Figure 9:**
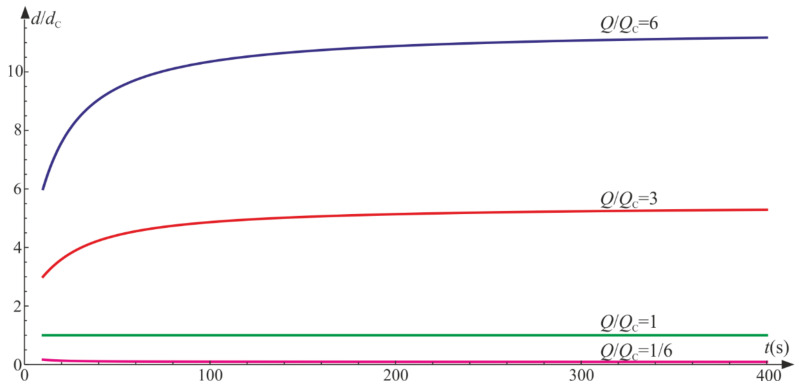
Uniaxial compression: ratio of damage parameters versus $Q/Q_{C}$.

The ratio $Q/Q_{C}$ governs the proportions of the isotropic and compressive damage. Based on Figure 9, if $Q/Q_{C}>1$, then throughout the process, $d/d_{C}>1$, and for $Q/Q_{T}<1$, $d/d_{C}<1$. In the case of $Q=Q_{C}$, a balanced $d\left(t\right)=d_{C}\left(t\right)$ exists as a result of the symmetry of system defined by Equation (75) with reference to the sought functions.

**Figure 10:**
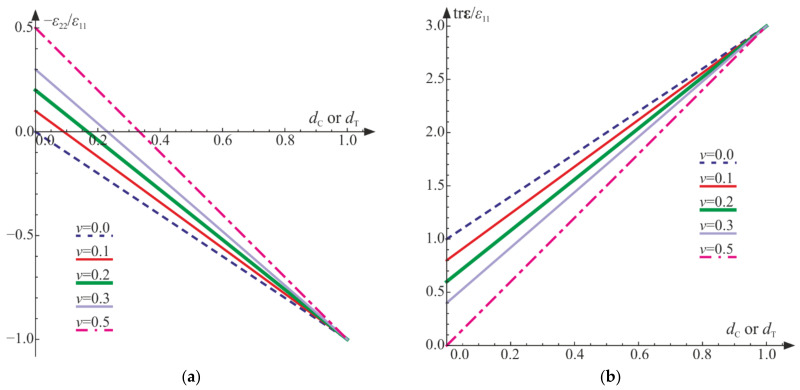
Uniaxial compression and uniaxial tension for various initial values of Poisson’s ratio ν: (**a**) generalized Poisson’s ratio versus damage parameters; (**b**) scaled trace of the strain tensor versus damage parameters.

During the loading process, the relative change of volume (the trace of the strain tensor) decreases, initially slowly, then rapidly. As shown above, for both the uniaxial tension and the uniaxial compression, the generalized Poisson’s ratio $-\varepsilon_{22}/\varepsilon_{11}$ can be either negative or positive (Figure 10a), but $\mathrm{tr}\varepsilon /\varepsilon_{11}$ is always non-negative (Figure 10b). As a result, the phenomenon of dilation, present in concrete and rocks, cannot be described properly only by this model.

**Figure 11:**
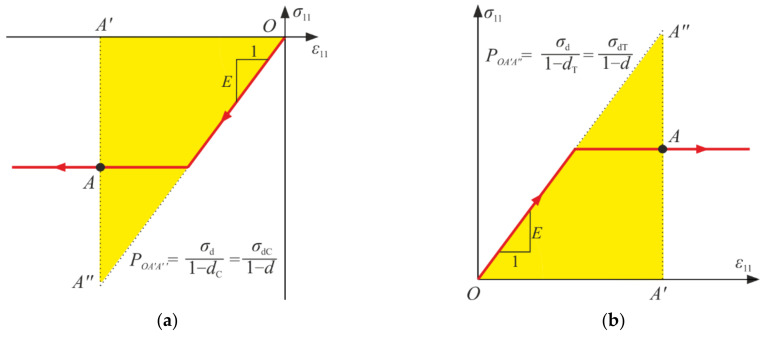
Graphical interpretation of the generalized stresses for point A of the loading path in the uniaxial compression (**a**) and the uniaxial tension (**b**).

As mentioned in Section 3, the generalized stresses are some parts of the strain energy, so they can be interpreted as proper areas (triangles QA′A″) connected to σ−ε curves, which is depicted in Figure 11. They can be associated to the strain energies of material in the undamaged state.

The actual behavior of material can include hardening/softening. For such a case, a preliminary model prediction is compared with experimental data for concrete [25,42,43]. Equations (65)–(74) remain true, but the system described by Equation (75) changes as follows:

(77) $$\left\{\begin{array}{c} \left(1-d\left(t\right)\right)\left(1-d_{C}\left(t\right)\right)=\frac{t_{0}}{t}\frac{\sigma_{C}\left(t\right)}{\sigma_{C0}},\;\;\;\;\;\;\;\\ \dot{d}\left(t\right)\left(1-d\left(t\right)\right)Q_{C}={\dot{d}}_{C}\left(t\right)\left(1-d_{C}\left(t\right)\right)Q,\\ d_{C}\left(t_{0}\right)=0,\;\;d\left(t_{0}\right)=0.\;\;\;\;\;\; \end{array}\right.$$

$\sigma_{C0}$ denotes an initial damage limit in the uniaxial compression, and $t_{0}=\sigma_{C0}/Ea$. The current damage limit $\sigma_{C}$ is assumed as the following function:

(78) $$\sigma_{C}\left(t\right)=\sigma_{C0}-\left(f_{C}-\sigma_{C0}\right)\left[\frac{d_{C}\left(t\right)}{d_{0}}-2\right]\frac{d_{C}\left(t\right)}{d_{0}}.$$

**Figure 12:**
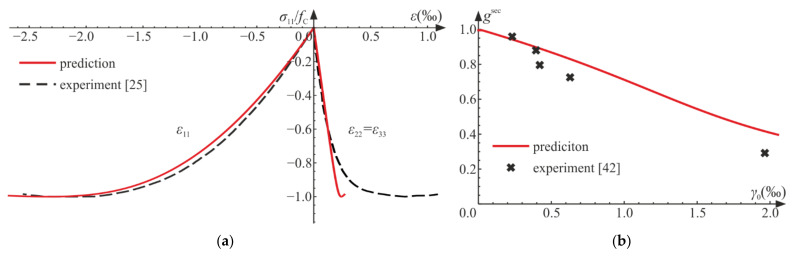
Uniaxial compression test—comparison with experimental data [25,42]: (**a**) stress versus strains; (**b**) scaled secant Kirchhoff modulus $g^{\sec}$ versus octahedral shear strain $\gamma_{0}=q/\sqrt{3}$.

The maximum value of the damage limit $f_{C}$ is attained for $d_{0}$. The considered system is solved numerically using the NDSolve procedure of Wolfram Mathematica for E=31 GPa, ν=0.2, $\sigma_{C0}=1\;\mathrm{MPa}$, $f_{C}=32\;\mathrm{MPa}$, $d_{0}=0.08$, and $a=0.0001\;s^{-1}$, Q=5, $Q_{C}=1$. The damage limit is attained at $t_{0}=0.323\;s$. Figure 12a shows the $\sigma_{11}-\varepsilon_{11}$ and $\sigma_{11}-\varepsilon_{22}$ curves in comparison with experimental results [25]. The material constants and the function describing $\sigma_{C}\left(t\right)$ in Equation (78) were assumed on the basis of the relationship between the axial strain and stress, that is, $\sigma_{11}-\varepsilon_{11}$.

The process of deterioration begins at $\sigma_{C0}=0.03\;f_{C}$. The experimental results taken from the literature [25,42,43] indicate that both bulk and shear moduli start to decrease for fairly low levels of loading. Let us introduce the following dimensionless secant moduli:

(79) $$\begin{array}{l} k^{\sec}=\frac{K_{C}}{K}=\frac{\frac{E\left(1-d_{C}\right)}{3\left[1-2\nu +2\;d_{C}\left(1+\nu \right)\right]}}{\frac{E}{3\left(1-2\nu \right)}}=\frac{\left(1-2\;\nu \right)\left(1-d_{C}\right)}{1-2\nu +2\;d_{C}\left(1+\nu \right)},\\ g^{\sec}=\frac{G^{\sec}}{G}=\frac{\frac{E\left(1-d\right)}{2\left(1+\nu \right)}}{\frac{E}{2\left(1+\nu \right)}}=1-d. \end{array}$$

**Figure 13:**
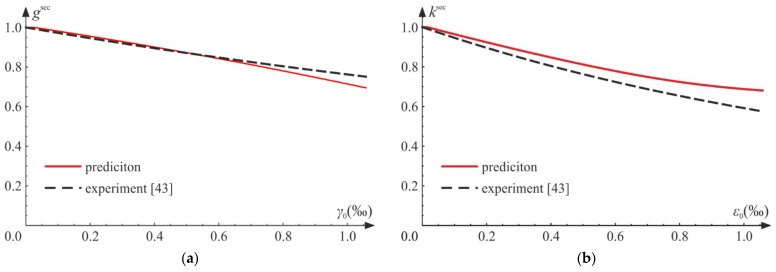
Uniaxial compression test: comparison of experimental [43] and numerical results: (**a**) scaled secant Kirchhoff modulus $g^{\sec}$ versus octahedral shear strain $\gamma_{0}=q/\sqrt{3}$; (**b**) scaled secant bulk modulus $k^{\sec}$ versus octahedral hydrostatic strain $\varepsilon_{0}=p/\sqrt{3}$.

A comparison of the scaled current shear moduli $g^{\sec}$ is presented in Figure 12b. In experiments, the shear modulus decreases with growing strain [42], which is reflected in numerical results. In Figure 13, we compare the scaled moduli with experimental regression curves obtained in [43]. In Figure 13a, values of $g^{\sec}$ are presented, and for the considered strain range, the compatibility of results is good. The agreement of predictions of the scaled bulk moduli $k^{\sec}$ and experiments seems satisfactory (see Figure 13b). Both functions, $k^{\sec}$ and $g^{\sec}$, diverge from the results adopted from the literature for higher levels of loading. This is due to the fact that after reaching some level of loading, plastic strains strongly influence the behavior of the material and interact with the advancing damage, which is not included in the considered model. The lateral strains (Figure 12a) obtained numerically are in good agreement with experimental results, until plastic dilation starts to play a major role in the process.

### 4.3. Uniaxial Cyclic Compression–Tension

Now, we investigate the material’s behavior in a uniaxial cyclic test. The primary focus is the transitions from tension to compression and from compression to tension and the changes of stiffness connected to them. All the governing equations for the uniaxial tests have already been given in Section 4.1 and Section 4.2 . Therefore, we do not describe the process in detail. Instead, we concentrate on the graphical representations of the obtained results.

**Figure 14:**
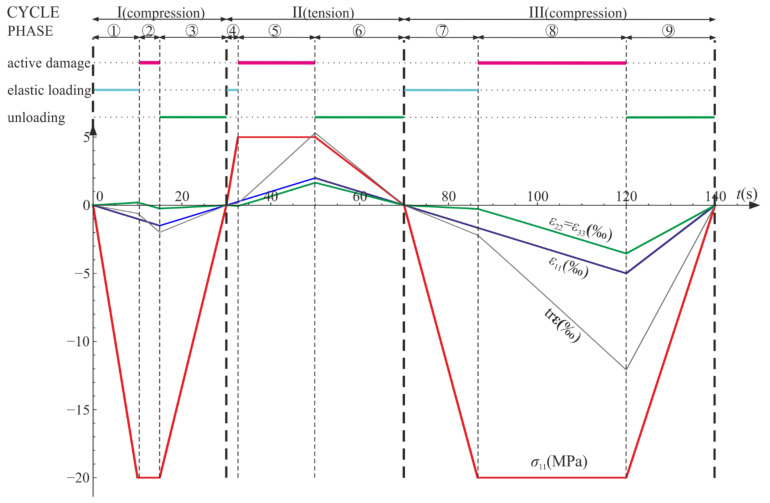
Uniaxial cyclic compression–tension: $\sigma_{11}$, $\varepsilon_{11}$, $\varepsilon_{22}=\varepsilon_{33}$, and trε with respect to time. Numbers ①–⑨ denote phases described in the text.

**Figure 15:**
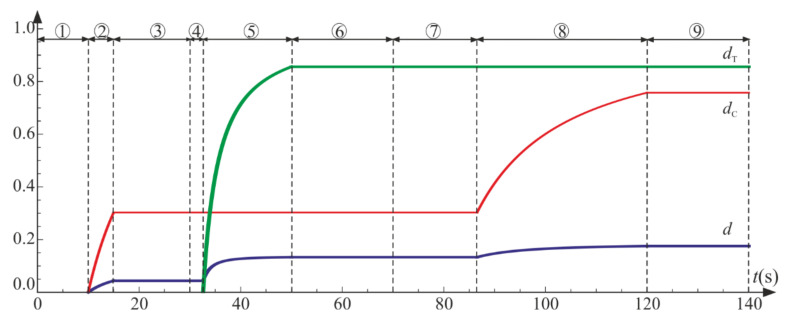
Uniaxial cyclic compression–tension: damage parameters with respect to time. Numbers ①–⑨ denote phases described in the text.

**Figure 16:**
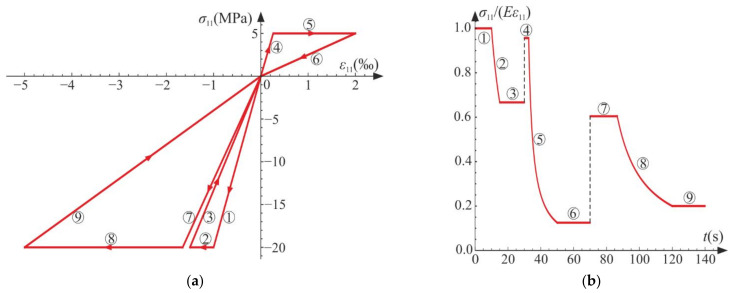
Uniaxial cyclic compression–tension: (**a**) axial stress versus axial strain; (**b**) degradation of the secant stiffness in time. Numbers ①–⑨ denote phases described in the text.

The following cycles and phases are considered (see also Figure 14, Figure 15 and Figure 16, where phases are denoted as ①–⑨):

- Cycle I: the uniaxial compression including: phase 1 (elastic loading for $\sigma_{11}>-\sigma_{C}$), phase 2 (loading with active damage process, $\sigma_{11}=-\sigma_{C}$), phase 3 (elastic unloading up to $\sigma_{11}=0$);
- Cycle II: the uniaxial tension including: phase 4 (elastic loading for $\sigma_{11}<\sigma_{T}$), phase 5 (loading with active damage process, $\sigma_{11}=\sigma_{T}$), phase 6 (elastic unloading to $\sigma_{11}=0$);
- Cycle III: the uniaxial compression including: phase 7 (elastic loading for $\sigma_{11}>-\sigma_{C}$), phase 8 (loading with active damage process, $\sigma_{11}=-\sigma_{C}$), phase 9 (elastic unloading to $\sigma_{11}=0$).

According to the assumed strain driven loading program, $\sigma_{11}$ stress can change its sign. The strain and stress tensors are diagonal in accordance with Equations (50) and (64). A piecewise linear program of axial strain $\varepsilon_{11}$ is assumed to be similar to Equations (56) and (69). The direct strain $\varepsilon_{11}$, lateral strains $\varepsilon_{22}=\varepsilon_{33}$, and axial stress $\sigma_{11}$ are bound by either Equation (54) or Equation (68). Functions of the strains and the stress with respect to time are shown in Figure 14. The results are delivered using Wolfram Mathematica for E=20 GPa, ν=0.2, $\sigma_{C}=20\;\mathrm{MPa}$, $\sigma_{T}=5\;\mathrm{MPa}$, and Q=1, $Q_{T}=Q_{C}=6$.

The axial stress and strain change piecewise linearly throughout the process, while the lateral components develop nonlinearly when the degradation advances (phases 2, 5, and 8). The volume change (trε) is always positive (expansion) for the tension (cycle II) and always negative (contraction) for the compression (cycles I and III).

**Figure 17:**
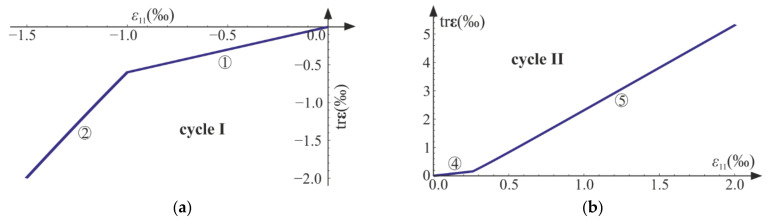
Volumetric strain trε versus axial strain $\varepsilon_{11}$: (**a**) uniaxial compression, cycle I; (**b**) uniaxial tension, cycle II. Numbers ①, ②, ④ and ⑤ denote phases described in the text.

The compressive damage parameter $d_{C}$ increases in phases 2 and 8, while the tensile damage parameter $d_{T}$ evolves only in phase 5. The isotropic damage parameter d grows whenever the degradation process is active (phases 2, 5, 8) (compare in Figure 15). This affects the slope of $\sigma_{11}-\varepsilon_{11}$ (Figure 16a) for the elastic loading and unloading. That is, the secant stiffnesses change as follows:

(80) $$\frac{E^{\sec,T}\left(t\right)}{E}=\frac{\sigma_{11}\left(t\right)}{E\;\varepsilon_{11}\left(t\right)}=\left(1-d_{T}\left(t\right)\right)\left(1-d\left(t\right)\right)\;\mathrm{for}\;\mathrm{uniaxial}\;\mathrm{tension},\;\frac{E^{\sec,C}\left(t\right)}{E}=\frac{\sigma_{11}\left(t\right)}{E\;\varepsilon_{11}\left(t\right)}=\left(1-d_{C}\left(t\right)\right)\left(1-d\left(t\right)\right)\;\mathrm{for}\;\mathrm{uniaxial}\;\mathrm{compression},$$

which is shown in Figure 16b. Let us highlight that the abrupt changes of the stiffness occur when passing through $\varepsilon_{11}=0$ (p=0 and ξ=0), that is, for transitions from phase 3 to phase 4 (compression to tension) and from phase 6 to phase 7 (tension to compression). The discontinuity of stiffness in the graph is caused by the switch between $d_{T}$ and $d_{C}$. In an arbitrary test, they cannot increase simultaneously in the regarded point, which ensures the clear distinction between tension and compression. The isotropic damage parameter d connects those two states: the tensile secant stiffness $E^{\sec,T}$ decreases during the uniaxial tension due to the lowering of $\left(1-d\right)$, and analogously, $E^{\sec,C}$ drops during the uniaxial compression. Therefore, the damage parameter d captures the history of damage that occurred before a certain instant of time but still affects the actual state of material; even the sign of loading is switched. In Figure 16b, there is an apparent difference between the secant stiffness for the first unloading in the uniaxial compression (phase 3) and the elastic loading in the next compression cycle (phase 7). It is due to the change of d during tension (cycle II). The absolute value of volume change (trε) always grows during loading phases, as pictured in Figure 14 and Figure 17.

### 4.4. Pure Shear

For the pure shear, the strain and stress tensors and their invariants are as follows:

(81) $$\varepsilon =\varepsilon_{12}\left(b_{1}⊗b_{2}+b_{2}⊗b_{1}\right),\;\sigma =\sigma_{12}\left(b_{1}⊗b_{2}+b_{2}⊗b_{1}\right)\;\mathrm{for}\;\sigma_{12}\geq 0,$$

(82) $$\xi =0,\;r=\sqrt{2}\;\sigma_{12},\;\theta =\pi /6,$$

(83) $$p=0,\;q=\sqrt{2}\;\varepsilon_{12}=\frac{1+\nu }{\;E\;\left(1-d\right)}r$$

and as a result:

(84) $$\varepsilon =\frac{1+\nu }{E\left(1-d\right)}s\;\mathrm{and}\;\varepsilon_{12}=\frac{1+\nu }{E\left(1-d\right)}\sigma_{12}.$$

We define the strain-driven loading program:

(85) $$\varepsilon_{12}\left(t\right)=a\;t,\;a>0.$$

Until the damage limit for the pure shear $\sigma_{S}$ is reached, the material’s response to loading is purely elastic in the form:

(86) $$\sigma_{12}\left(t\right)=\frac{E\left(1-d\right)}{1+\nu }a\;t,\;d\left(t\right)=0,\;d_{T}\left(t\right)=0,\;d_{C}\left(t\right)=0\;\mathrm{for}\;t<t_{0}=\frac{\sigma_{S}\left(1+\nu \right)}{E\;a\;}.$$

Further loading leads to degradation. The damage condition (Equation (34)) reduces to:

(87) $$\sigma_{12}-\sigma_{S}=0.$$

Using Equation (24), the generalized stresses are obtained:

(88) $$\sigma_{d}^{}=\frac{E}{1+\nu }\varepsilon_{12}^{2},\;\sigma_{dT}^{}=0,\;\sigma_{dC}^{}=0,$$

and subsequently, the evolution laws (Equation (32)) take the form:

(89) $$\dot{d}=2\lambda \;Q\frac{E}{1+\nu }\varepsilon_{12}^{2},\;{\dot{d}}_{T}=0,\;{\dot{d}}_{C}=0.$$

As a result, $d_{T}\left(t\right)=0$ and $d_{C}\left(t\right)=0$; thus only the isotropic damage parameter evolves. To get $d\left(t\right)$ for $t\geq t_{0}$, it suffices to solve the first relationship of Equation (86), taking into consideration the damage condition from Equation (87):

(90) $$d\left(t\right)=1-\frac{\left(1+\nu \right)\sigma_{S}}{Ea\;t}.$$

The graphs are obtained for E=20 GPa, ν=0.2, $\sigma_{S}=5\;\mathrm{MPa}$, and $a=0.00001\;s^{-1}$. Let us notice that we do not need the value of Q for the investigated case. The ratios $Q/Q_{T}$ and $Q/Q_{C}$ govern the proportions of suitable damage parameters ($d/d_{T}$ and $d/d_{C}$) for tests with coinciding evolutions of two damage parameters. The absolute values of Q, $Q_{T}$, and $Q_{C}$ do not have a meaning of their own, so it is reasonable to set Q=1 and control the processes only through the ratios.

**Figure 18:**
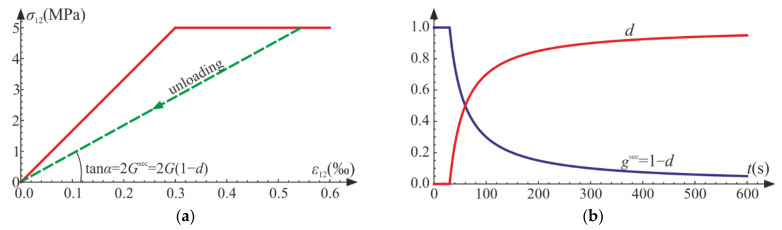
Pure shear: (**a**) strain versus stress; (**b**) evolution of damage parameter d and the scaled shear modulus $g^{\sec}=1-d$.

**Figure 19:**
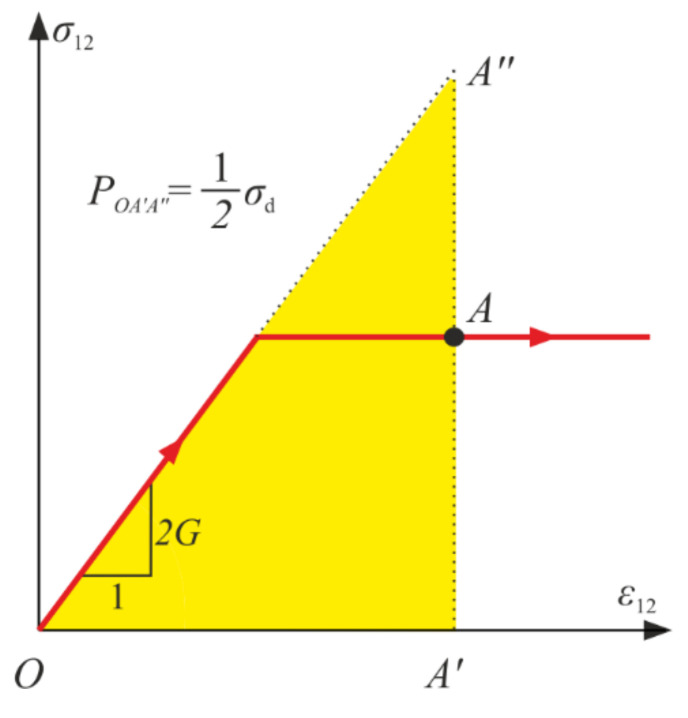
Graphical interpretation of the generalized stress $\sigma_{d}$ in pure shear for point A.

As depicted in Figure 18a, after the damage onset, the secant Kirchhoff modulus decreases compared with the initial value. The reduction is by factor $\left(1-d\right)$, so d can be directly interpreted in the pure shear experiment. When d→1, the failure occurs—the secant stiffness tends toward zero (compare in Figure 18b). Additionally, for the pure shear the generalized stress $\sigma_{d}$ is the doubled strain energy of the virgin material (see Figure 19).

## 5. Discussion

In the proposed model, three damage parameters, d, $d_{T}$, and $d_{C}$, are used. In any active damage process, the isotropic damage parameter changes; that is, $\dot{d}\neq 0$. The isotropic damage parameter d is introduced in a standard way (see in [2,3]), multiplying the Helmholtz energy (strain energy) by $\left(1-d\right)$, which can be directly caught in the pure shear test as a scaling factor for the stiffness of a damaged material. It can also be interpreted as a percent of a specimen’s area that underwent complete failure and thus does not carry any loading. The parameter d affects both the volumetric (bulk) and the distortional part of energy. Its evolution is associated with all stress states that satisfy the assumed damage condition.

On the contrary, two remaining parameters describe damage separately—evolution of $d_{T}$ excludes the simultaneous growth of $d_{C}$ and vice versa. They apply only to the strain (stress) states that have nonzero isotropic parts. The reason for including the damage parameters $d_{C}$ and $d_{T}$ into the model is to split the material’s response in two cases, so the stiffnesses in compression (p<0, ξ<0) and tension (p>0, ξ>0) can be governed individually. The parameters are introduced in such a way that they have clear interpretations in the uniaxial tests: their values control the current stiffnesses of the damaged material and establish unambiguous relationships between the axial and the lateral components of the strain tensor.

Why use three parameters instead of one or two? Using one parameter, d allows for describing only isotropic damage. This is not the realistic case for concrete and rocks, which behave in a more complex way. There is a crucial difference between tension and compression. For those materials, failure in tension is brittle and sudden, and experimental results show a narrow zone of softening [13,15,30]. For compression, the deterioration develops slowly, distinct yielding and hardening are observed, and the failure occurs through crushing [13,30]. The damage (and yield) limits for the uniaxial tension and compression are markedly different. Thus, we need at least two variables to describe the damage process. Models with two damage parameters, in particular the most used concrete damaged plasticity of Abaqus [36], are suitable for describing this dissimilarity but usually use, directly or indirectly, a split of stress or strain tensors into negative and positive parts, which entails differentiability problems. Using three damage parameters reduces this problem: the first derivative of the Helmholtz energy with respect to strain tensor is continuous (the relation between σ and ε is always unambiguous). The second derivative (the tensor of tangent stiffness) is not determined for p=0, but it can be improved by a simple regularization.

**Figure 20:**
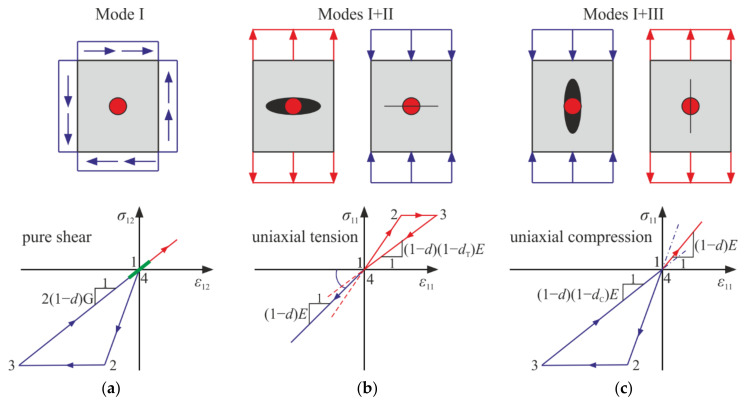
Modes of damage development in the considered model. Presented cycles of loading start at the natural state of material. (**a**) Mode I: isotropic damage for pure shear experiment; (**b**) modes I and II: a cycle of uniaxial tension and transition to uniaxial compression; (**c**) modes I and III: a cycle of uniaxial compression and transition to uniaxial tension.

Based on the analyses carried out in Section 4, let us make an attempt to associate the variables d, $d_{C}$, and $d_{T}$ with physical fracture phenomena. For the considered model, there are three damage modes (mechanisms) shown schematically in Figure 20 (compared with two modes described by Ortiz [33]). Mode I, connected to parameter d, is the isotropic damage as represented in Figure 20a. Simultaneously, depending on the sign of trε (trσ), one of two remaining modes can happen within a material point. In uniaxial tension, the fracture usually occurs in a plane perpendicular to the direction of subjected loading, while for the uniaxial compression, a specimen breaks either parallel or at a slight angle to the loading direction [27,28,31]. Mode II, associated with $d_{T}$, mirrors the evolution of cracks resulting from the extension coincident with the tensile loading’s direction. An increase in $d_{T}$ is represented by a black ellipse, while a simultaneous increase in d is depicted by a red circle within the ellipse in Figure 20b. During unloading with material stiffness $\left(1-d\right)\left(1-d_{T}\right)E$, those cracks partially close, which can be interpreted as regaining the stiffness up to $\left(1-d\right)E$ when the loading is changed to compressive. In Figure 20b, this process is represented by a black ellipse (mode II—opening) contracting to a line (mode II—closure). The red circle is a symbol of isotropic degradation (mode I) that transfers to compressive states. Mode III, associated with the evolution of $d_{C}$, represents crushing, which is a situation where the compressive loading causes cracks to open in planes parallel to the loading’s direction. Additionally, in this case, the damage can be partly “undone” by switching to tension as a result of rearrangement (wedging) of grains, so mode III is accompanied by mode I, as shown in Figure 20c. In general, the isotropic damage parameter d representing mode I plays the role of a restitution parameter, compare with the concrete damaged plasticity model in Abaqus [12,35,36]. It preserves the memory of the former damage when the sign of loading is changed, which is indispensable in the analyses of cyclic loadings.

Any of the three damage parameters, d, $d_{T}$, and $d_{C}$, can be easily removed from the model. It suffices to eliminate in Equation (16) the unwanted term connected to one of the coefficients, Q, $Q_{T}$, or $Q_{C}$, and exclude the damage parameter from energy (Equation (12)) related to it, but their presence seems to be reasoned when comparing with the experimental evidence, at least for concrete. It is operative to set Q=1 and establish the ratios $Q/Q_{T}$ and $Q/Q_{C}$. This should be performed by comparing the results of numerical calculations for the model with experimental data. That is, having $\varepsilon_{11}$, $\varepsilon_{22}=\varepsilon_{33}$, and $\sigma_{11}$ from tests, we can use relationships ins Equations (63), (76) and (90) to estimate $d\left(t\right)$, $d_{T}\left(t\right)$, and $d_{C}\left(t\right)$ and compare them with the functions received using the considered model. This allows for determining the appropriate levels of the ratios $Q/Q_{T}$ and $Q/Q_{C}$.

## 6. Conclusions

In the paper, a thermodynamically consistent model of an elastic damaged material is proposed. The emphasis is placed on the Helmholtz energy, which is not a quadratic form of the elastic strain tensor. It is required that the function enables a sharp distinction between tensile and compressive states, typical for quasibrittle materials. Compared with the functions present in the literature, the unquestioned advantage of the considered Helmholtz function is its differentiability, which results from omitting the split of the strain (stress) tensor into positive and negative parts. Still, introduction of three damage parameters allows for describing different modes of cracking development. Unfortunately, the assumed energy has an indeterminate second derivative with respect to ε when trε=0. This problematic flaw in the finite element calculations can be dealt with effectively using a regularization parameter in the tensor of tangent stiffness described by Equation (46).

The presented model is reasonably flexible. The introduction of three parameters in the definition of the dissipation function allows for effectively maneuvering between modes of damage evolution. Due to the proposed dissipation potential, it is possible to obtain any convex damage surface. A selection of damage function would influence the consistency condition, the damage multiplier, and the form of the tensor of tangent stiffness. However, the damage condition given in Equation (34) used in this paper possesses all the features required for concrete. Undoubtedly, the existence of the closed-form calibration formulae (Equations (35)–(37)) is an asset to the model. The evolution laws are constructed so as to guarantee the proportionality of damage parameters’ rates and portions of energy, which is thermodynamically consistent and broadly accepted.

Throughout the text, we refer to the quasibrittle materials, especially concrete, but do not offer extensive comparison with experimental data. This is due to the fact that a full description of such materials requires including plasticity. Elastic damage models not only neglect permanent deformation but also fail to depict dilation. The shown Helmholtz energy and dissipation potential constitute a model of their own, but they can be incorporated in a more complex description of an elastic-plastic damaged material.

## Data Availability

Data sharing is not applicable to this article.
